# *Bacillus velezensis* mitigates chronic LPS-induced lung injury of broilers via microbiota-driven isoflavone production and NF-κB/PPAR-γ axis modulation

**DOI:** 10.1186/s40104-026-01391-1

**Published:** 2026-05-07

**Authors:** Xunbozan Zhang, Xuan Liu, Ye Xu, Dadan Liu, Ying Chen, Christopher Peterson Daniel, Ruixiao Mao, Liang Chen

**Affiliations:** State Key Laboratory of Animal Nutrition and Feeding, Key Laboratory of Animal Nutrition and Feed Science of Ministry of Agriculture and Rural Affairs, Institute of Animal Science, Chinese Academy of Agricultural Sciences, Beijing, 100193 China

**Keywords:** *Bacillus velezensis*, Broiler lung, Chronic inflammation, Isoflavone, Lipopolysaccharide, NF-κB

## Abstract

**Background:**

Chronic exposure to low‑dose lipopolysaccharide (LPS) in poultry farming environments induces persistent respiratory inflammation, resulting in lung injury and impaired growth performance in broilers. *Bacillus velezensis* (BV) is a probiotic with known antibacterial and immunomodulatory activities, yet its role in respiratory health remains poorly understood. This study aimed to assess the potential benefits of BV in alleviating chronic pneumonia triggered by LPS in broilers and to clarify its mechanistic pathways.

**Results:**

A chronic LPS intratracheal instillation model was established, comprising control, LPS, and BV + LPS groups. BV supplementation significantly ameliorated LPS‑induced growth impairment (*P* < 0.05), inhibited the synthesis of key inflammatory mediators, and mitigated oxidative stress in serum and bronchoalveolar lavage fluid (*P* < 0.05). Integrated multi‑omics analyses revealed that BV remodeled the pulmonary microbiota, enriching isoflavone‑metabolizing taxa including *Blautia* and unclassified *Lachnospiraceae* (*P* < 0.05), which was associated with elevated pulmonary concentrations of daidzein, genistein, and glycitein (*P* < 0.05). Transcriptomic together with molecular analyses revealed that BV enhanced the activation of PPAR‑γ while attenuating NF‑κB pathway activity, thereby reducing the expression of genes associated with inflammation (*P* < 0.05). In vitro, experiments showed that daidzein and genistein inhibited cellular inflammatory responses through PPAR-γ signaling. BV culture supernatant directly suppressed NF‑κB/NLRP3 inflammasome activation in chicken HD11 macrophages, reduced intracellular reactive oxygen species (ROS) generation, and shifted macrophage polarization toward an anti‑inflammatory phenotype (*P* < 0.05).

**Conclusion:**

These findings demonstrate that BV alleviates LPS-induced chronic pneumonia through two complementary pathways, as it remodels the pulmonary microbiota to enhance isoflavone metabolism and thereby suppress inflammation, while its own metabolites also directly inhibit inflammatory signaling. This study provides new insight into probiotic-based interventions for respiratory health in livestock.

**Supplementary Information:**

The online version contains supplementary material available at 10.1186/s40104-026-01391-1.

## Introduction

Lipopolysaccharide (LPS), a major structural component of the cell wall of Gram-negative bacteria, is a potent activator of the host innate immune response. In modern high-density livestock and poultry farming environments, airborne microbial aerosols often contain significant levels of LPS [1], posing a potential threat to the respiratory health of animals and farm workers [2–4]. The study confirmed that long-term exposure to low-dose LPS can induce a sustained state of immune stress in broilers, while high doses can trigger acute lung injury and systemic inflammatory responses [5]. Therefore, identifying effective strategies to mitigate the decline in broiler performance caused by aerosolized LPS in farming environments is of substantial practical importance [6].

With the progressive restriction of antibiotic use in animal feed, there is growing interest in developing safe and efficient alternatives, such as enzymes, probiotics, and plant extracts [7]. Among the various probiotics, *Bacillus* species have gained attention due to their spore-forming capability, which ensures better survival in the gastrointestinal tract of livestock, and their ability to produce beneficial metabolites and antimicrobial compounds [8]. The *Bacillus velezensis* (BV) strain used in this study was isolated from the intestine of Jinhua pigs. Preliminary laboratory studies indicated that this strain exhibits significant antimicrobial activity and strong tolerance to various pH conditions, as well as simulated gastric and intestinal fluids.

BV was first isolated in 1999 and formally named in 2005 [9]. It is an aerobic, spore-forming, and antimicrobial-safe probiotic widely used in livestock and poultry production to improve intestinal health and immune function. Previous studies have shown that dietary BV supplementation significantly reduces intestinal barrier injury caused by *Salmonella* Typhimurium in mice, modulates cytokine expression, and optimizes gut microbiota structure [10]. In broilers, BV supplementation mitigates the negative effects of LPS on intestinal cytokines, antioxidant capacity, and barrier function [11]. Furthermore, feeding broilers with BV-fermented corn flour not only reduces mortality but also optimizes gut microbiota composition, with effects superior to some antibiotics [12].

Current research on the effects of LPS on broiler lungs is relatively limited and predominantly focuses on acute, short-term exposure models. However, chronic inflammatory stimulation can induce tolerance in organisms. The common scenario in farming environments—low-dose, chronic LPS exposure—likely involves significant differences in multiple signaling pathways and immune response patterns compared to acute challenge models. Furthermore, although *Bacillus subtilis* has been extensively studied for its various applications in animal production, research on BV is less abundant and has primarily concentrated on improving intestinal health. Reports on its role in the respiratory system, particularly regarding the prevention and control of pulmonary inflammation, are scarce. Given this context, this study aims to evaluate the efficacy of BV in alleviating LPS-induced chronic pulmonary inflammation in broilers and to elucidate the underlying molecular mechanisms, thereby providing a theoretical foundation for its application in the management of respiratory health in livestock and poultry.

## Materials and methods

### Animals, bacterial strain, and experimental design

One-day-old AA commercial broilers were obtained from Beijing Dafaun Poultry Breeding Co., Ltd. (Beijing, China). All experimental procedures were conducted in accordance with the guidelines for the care and use of laboratory animals and were approved by the Animal Protection Committee of the Institute of Animal Science, Chinese Academy of Agricultural Sciences (Approval No. IAS2025-161).

The BV strain used in the experiment was preserved in our laboratory. When the bacterial culture reached a concentration of 1 × 10^8^ CFU/mL, it was centrifuged at 3,000 × *g* to remove the supernatant, and the pellet was resuspended in an equal volume of sterile physiological saline. LPS was purchased from Sigma-Aldrich (St. Louis, MO, USA).

Broilers were reared in an environmentally controlled chamber from day 1 of age. The initial temperature was set at 36 °C with 50%–60% relative humidity. From d 1 to d 14, the temperature was lowered by 0.5 °C every other day, reaching 33 °C on d 7 and 30 °C on d 14. Feed and water were provided ad libitum throughout the experimental period. Broiler treatment dose and administration frequency were determined with reference to previous studies [13, 14]. Forty-five broilers were randomly allocated to three treatments (*n* = 15 per group; 3 cages × 5 broilers each): Sal group (0.5 mL saline), LPS group (0.25 mL 4 mg/mL LPS + 0.25 mL saline), and BV + LPS group (0.25 mL 1 × 10^8^ CFU/mL *B. velezensis* + 0.25 mL 4 mg/mL LPS). The total volume of the perfusate administered to each broiler across all groups was consistent at 0.5 mL. Tracheal instillations were conducted on d 14, 17, 20, 23, and 26. Body weight was measured on d 27, and broilers were slaughtered on d 28 after a 12-hour fast.

### Sample collection and analysis

Body weight was recorded on d 27 to assess growth performance. On d 28, all broilers were euthanized, and visceral organs were immediately collected and weighed to calculate relative organ indices.

Lung tissues were collected after slaughter. A portion was fixed in 4% paraformaldehyde, embedded, and sectioned for hematoxylin-eosin (H&E) staining. Histopathological alterations were examined and imaged under a light microscope. The remaining lung tissue was snap-frozen in liquid nitrogen and stored at −80 °C for subsequent RNA and protein extraction.

Bronchoalveolar lavage fluid (BALF) was collected under anesthesia by exposing and incising the trachea, followed by flushing the airway with sterile physiological saline (1 mL/100 g body weight). The lavage fluid was centrifuged at 3,000 × *g* for 10 min at 4 °C, and the supernatant was aliquoted and stored at −80 °C for further analysis.

Blood samples were obtained from the brachial vein, centrifuged at 3,000 × *g* for 10 min to separate serum, and stored at –80 °C. Whole blood was collected into EDTA-anticoagulated tubes, gently mixed, and immediately analyzed for hematological parameters using an automated hematology analyzer (PE-6800Vet, Prokan, China).

Commercial ELISA kits (Shanghai Jining Biotechnology Co., Ltd., China) were used to quantify immune-related markers (IgA) and inflammatory cytokines (IL-1β, IL-6, IL-8, IL-10, IL-17A, and TNF-α) in BALF and serum, as well as antioxidant indices, including total antioxidant capacity (T-AOC), malondialdehyde (MDA), superoxide dismutase (SOD), catalase (CAT), and glutathione peroxidase (GSH-Px).

### Cell culture and treatments

HD11 macrophages were cultured in RPMI-1640 medium supplemented with 10% FBS and 1% penicillin–streptomycin at 37 °C with 5% CO_2_ and used during the logarithmic growth phase. A CCK-8 assay (Solarbio, Beijing, China) was used to determine non-cytotoxic concentrations of BV supernatant (SN) and LPS. BV cultures were grown to OD_600_ = 1.5, and the supernatant was filtered (0.22 μm). Cells were treated with 0–20% (v/v) BV SN for 6 h, and viability was assessed at 450 nm. LPS was tested at 0–20 μg/mL, and optimal concentrations were selected accordingly. After 6 h of treatment, protein and mRNA expression were analyzed by Western blotting and RT-qPCR.

Genistein and daidzein were purchased from Solarbio, and GW9662 was purchased from MCE. Based on previous reports [15–17], these compounds were applied to cells at final concentrations of 10 μmol/L, 25 μmol/L and 10 μmol/L respectively. After 6 h of treatment, protein and mRNA expression were analyzed by Western blotting and RT-qPCR.

### 16S rRNA gene sequencing analysis

For pulmonary microbiome sequencing, lung tissue samples were collected from the Sal, LPS, and BV + LPS groups, with six biological replicates per group randomly selected from individual broilers.

DNA extraction, PCR amplification, and sequencing library preparation were performed by Majorbio Bio-Pharm Technology Co., Ltd. (Shanghai, China). Raw sequencing reads were processed using the UPARSE software package and clustered into operational taxonomic units (OTUs) at a 97% sequence similarity threshold, followed by removal of chimeric sequences to obtain representative OTU sequences. Taxonomic assignment of OTUs was conducted using the RDP Classifier algorithm against the SILVA 16S rRNA gene reference database, with a confidence threshold of 70%. All downstream bioinformatics analyses were performed on the Majorbio Cloud Platform, and raw sequencing data were deposited in the NCBI SRA database under accession number PRJNA1337892.

### Transcriptome analysis

Lung tissues were obtained for RNA isolation using the phenol–chloroform protocol. The cDNA library was constructed and sequenced by Majorbio Bio-Pharm Technology Co., Ltd. (Shanghai, China). All sequencing data were processed through the Majorbio Cloud Platform, and the dataset was submitted to the SRA under accession number PRJNA1343556.

### Metabolomics analysis

Non-targeted metabolomics analysis of lung tissues was performed at Majorbio Bio‑Pharm Technology Co., Ltd. (Shanghai, China) using a Thermo UHPLC Orbitrap Exploris 240 system (Thermo Fisher Scientific, USA). Samples were processed following standard protocols to obtain metabolite extracts. Metabolite identification was achieved by matching against the HMDB, Metlin, and Majorbio's proprietary databases. Statistical analyses were performed on the Majorbio Cloud Platform, and metabolites with VIP > 1 and *P* < 0.05 were considered significantly different.

### Quantitative real-time PCR (qRT-PCR)

Total RNA was extracted from lung tissues and HD11 cells using the TransZol Up Plus RNA Kit (TransGen Biotech, Beijing, China). RNA purity and concentration were assessed using a BioDrop-μLite spectrophotometer. cDNA was synthesized with gDNA removal using EasyScript One-Step SuperMix (TransGen Biotech, Beijing, China). Gene-specific primers were designed using NCBI Primer-BLAST (Table S1) and synthesized by Sangon Biotech (Shanghai, China). qRT-PCR was performed on an ABI Q7 Flex 96 T system using SYBR^®^ Premix Ex Taq™ II (Takara, Japan). Gene expression was calculated using the 2^−ΔΔCt^ method and *β‑actin* as the internal reference gene.

### Western blotting

Proteins were extracted from lung tissues and HD11 cells using RIPA buffer (Beyotime, Beijing, China) with PMSF. Concentrations were determined by BCA assay (Thermo Fisher, USA). Proteins were separated by SDS-PAGE and transferred to PVDF membranes (Bio-Rad, USA). After blocking with 5% non-fat milk for 1 h at room temperature, membranes were incubated with primary and HRP-conjugated secondary antibodies (listed in Table S2), followed by ECL detection (SUNBIOO, Beijing, China). Bands were visualized using a ChemiDoc imaging system (Servicebio, Wuhan, China) and quantified relative to β-actin using the system's built-in image analysis software.

### Statistical analysis

Phenotypic data, inflammatory cytokine levels, oxidative stress makers, and relative gene/protein expression data were analyzed by one-way analysis of variance (ANOVA) followed by Tukey's multiple comparison test using SPSS 24. Results were presented as mean ± standard error of the mean (SEM). Figures were generated using GraphPad Prism 10.1.2. Pearson correlation analyses were performed in RStudio 4.5.2, and results were presented as *r* values with significance levels. Statistical significance was set at *P* < 0.05.

## Results

### BV mitigates LPS-induced growth retardation and lung injury in broilers

**Fig. 1:**
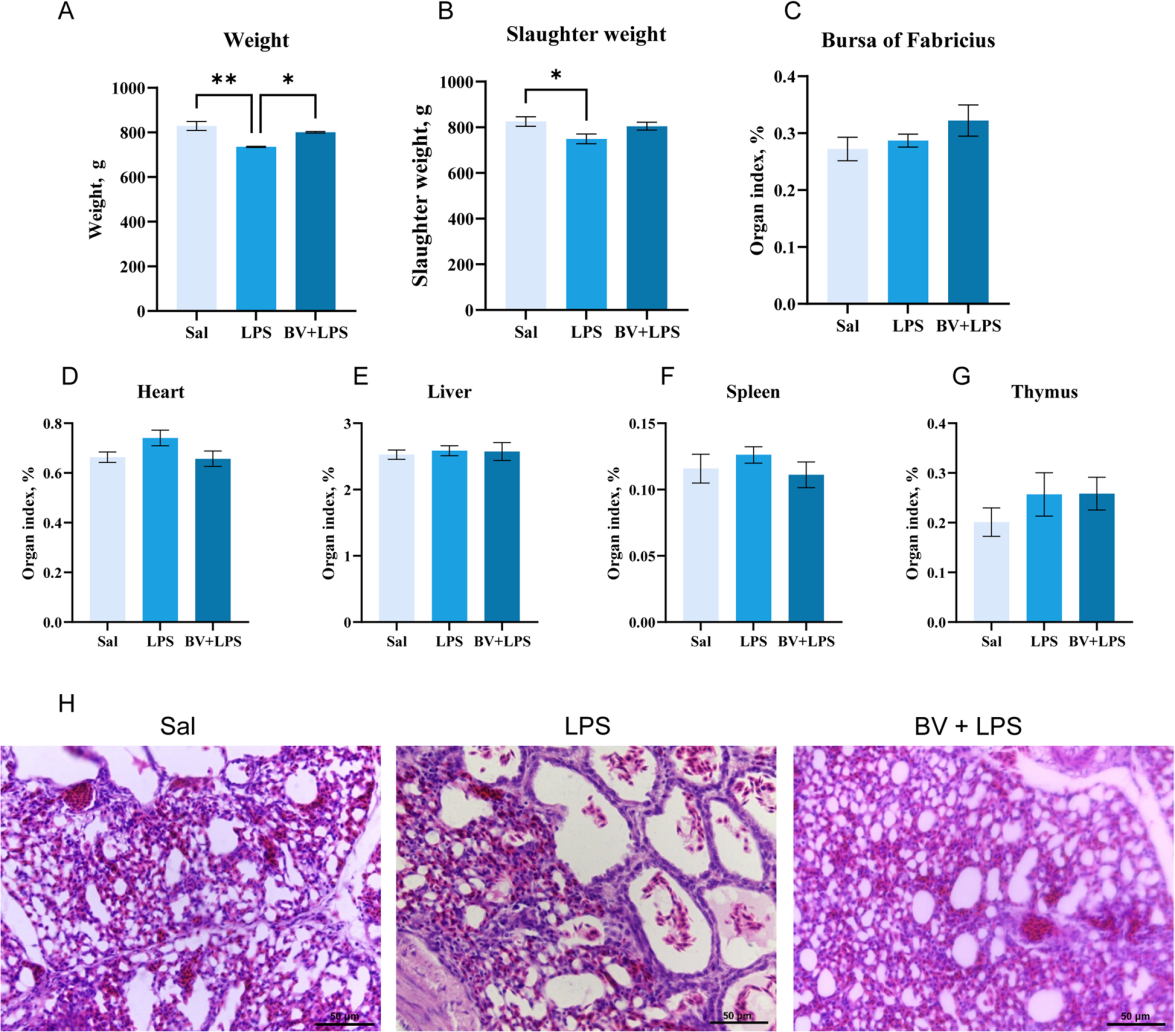
Effects of LPS challenge and *Bacillus velezensis* (BV) supplementation on growth performance, organ indices, and lung histopathology in broilers in three groups. **A** Body weight (*n* = 3). **B** Slaughter weight (*n* = 3). **C**–**G** Organ indices (*n* = 9). **H** Representative H&E-stained lung sections (scale bar = 50 μm). Data are presented as mean ± SEM. ^*^P < 0.05, ^**^P < 0.01

Intratracheal administration of LPS significantly decreased live and final body weights compared to the saline group (*P* < 0.05), whereas BV supplementation effectively reversed this growth suppression and restored performance to control levels (*P* < 0.05; Fig. 1A and B). Relative organ weights remained unchanged among the groups, suggesting that systemic organ development was largely unaffected by LPS insult (Fig. 1C–G). H&E staining revealed pronounced thickening of alveolar septa, dilation of alveolar spaces, and erythrocyte infiltration in the LPS group. These pathological changes were markedly alleviated in the BV + LPS group, with lung morphology closely resembling that of the Sal group (Fig. 1H).

### BV attenuates LPS-induced pulmonary and systemic inflammation and oxidative stress

**Fig. 2:**
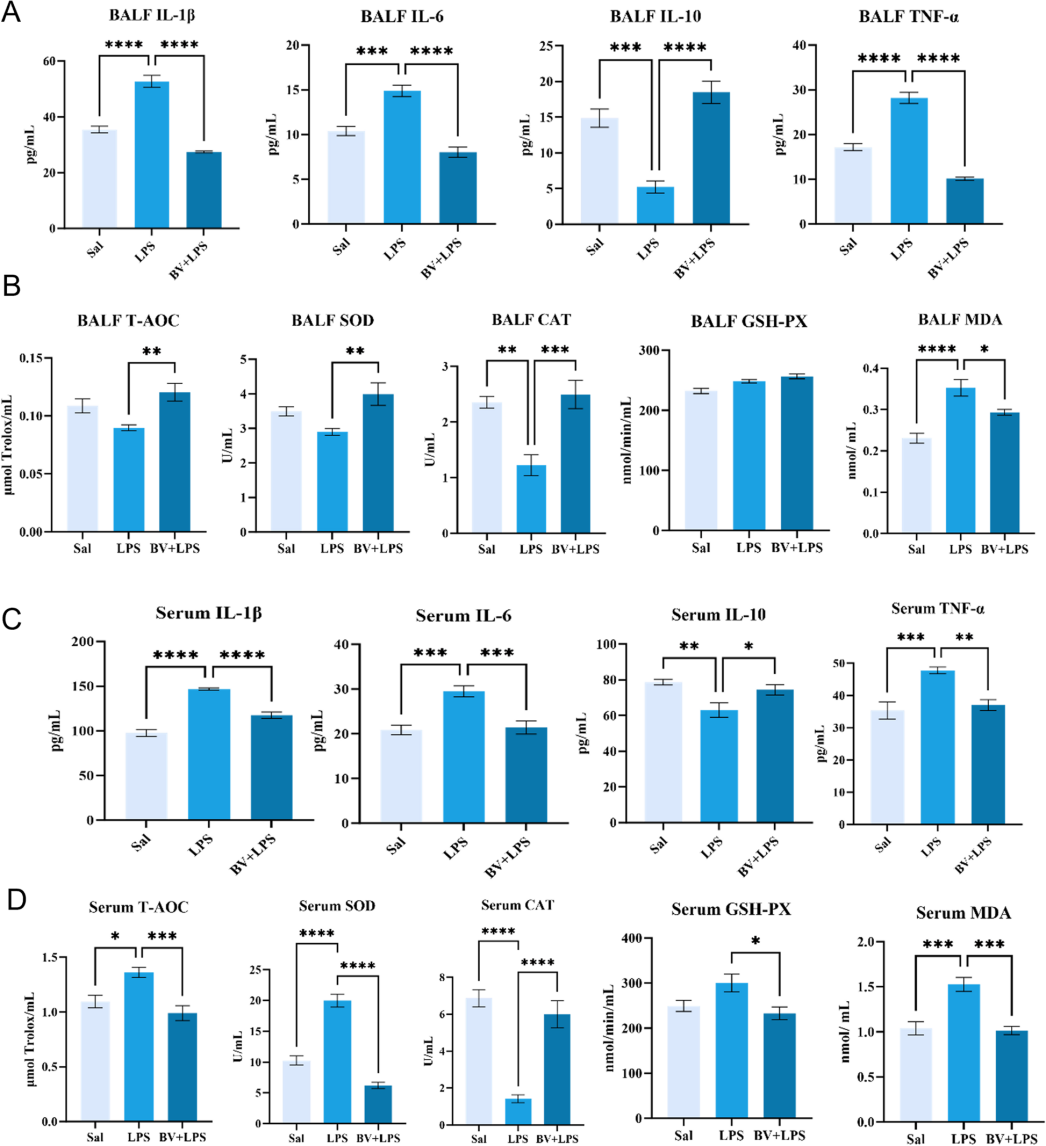
Effects of LPS exposure and BV supplementation on inflammatory cytokines and oxidative stress indices in serum and bronchoalveolar lavage fluid (BALF) (*n* = 6). **A** BALF cytokines. **B** BALF antioxidant and oxidative damage markers. **C** Serum cytokines. **D** Serum antioxidant and oxidative damage markers. Data are presented as mean ± SEM. ^*^P < 0.05, ^**^P < 0.01, ^***^P < 0.001, ^****^P < 0.0001

LPS exposure primarily triggered a pronounced inflammatory response in the lung. In BALF, pro‑inflammatory cytokines IL‑1β, IL‑6, and TNF‑α were markedly elevated (*P* < 0.05), whereas the anti‑inflammatory mediator IL‑10 was significantly suppressed, indicating a shift toward a pro‑inflammatory state. BV supplementation effectively reversed these alterations, restoring cytokine profiles toward baseline levels. Regarding oxidative stress in BALF, LPS administration induced a compensatory increase in antioxidant defense markers, accompanied by a significant rise in MDA and a decline in CAT activity (*P* < 0.05), reflecting redox imbalance. BV supplementation normalized both antioxidant enzyme activities and MDA levels, suggesting mitigation of pulmonary oxidative injury (Fig. 2A and B).

In serum, LPS exposure significantly increased levels of IL-1β, IL-6, and TNF-α, and decreased IL-10 (*P* < 0.05). BV reversed these changes, restoring cytokine levels toward those in the Sal group. Similarly, serum oxidative stress parameters showed elevated antioxidant defenses (T‑AOC, SOD, GSH‑Px) alongside increased MDA and reduced CAT activity (*P* < 0.05). BV supplementation improved antioxidant status and reduced lipid peroxidation levels. No significant changes were observed in hematological parameters across treatment groups (Table S3), indicating that LPS induced systemic inflammatory markers without causing systemic toxicity (Fig. 2C and D).

### BV supplementation reshapes pulmonary microbiota composition in LPS-challenged broilers

**Fig. 3:**
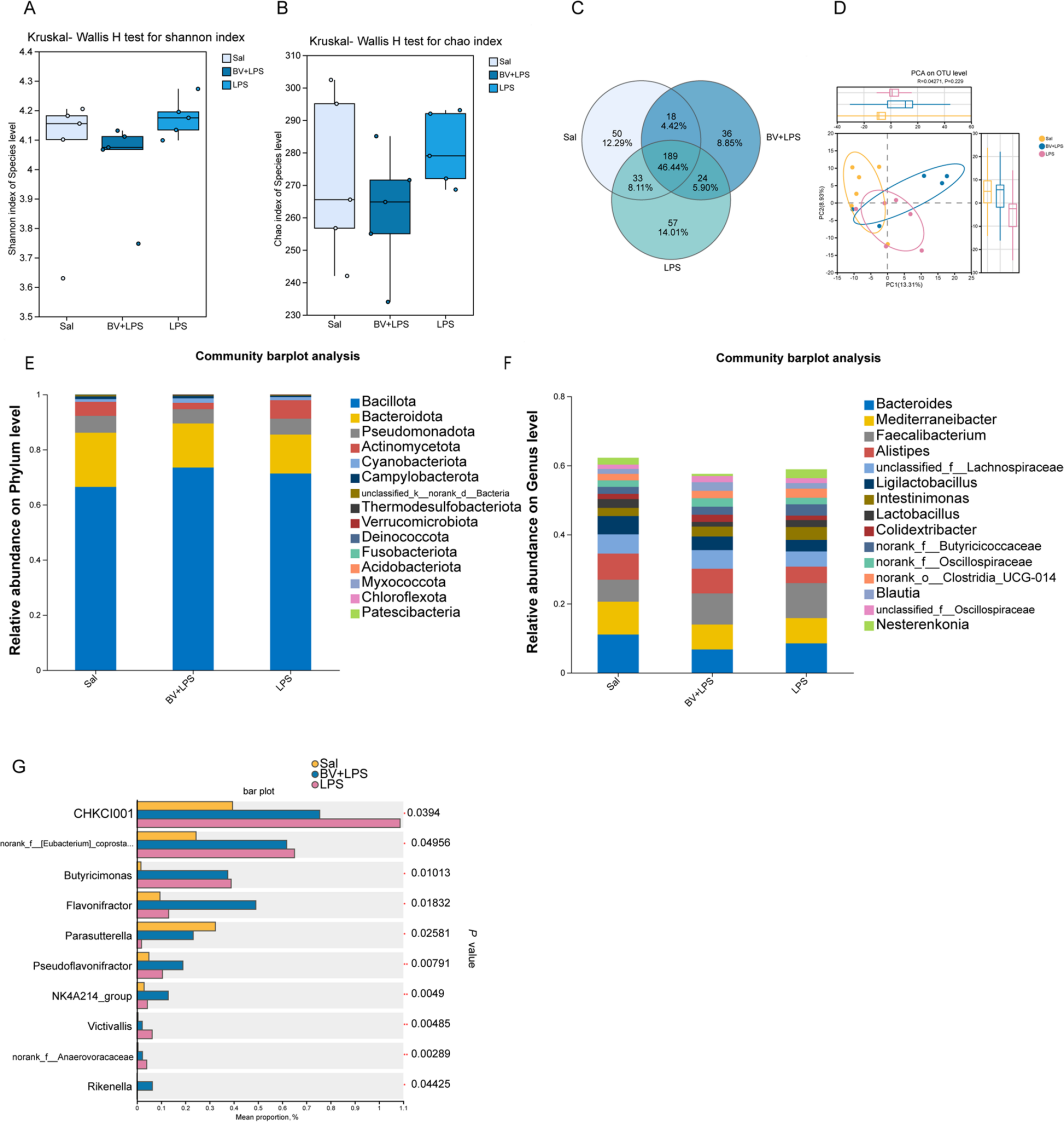
Pulmonary microbiota diversity (*n* = 6). **A** Shannon index analysis. **B** Chao index analysis. **C** Venn diagram. **D** Principal component analysis. **E** Phylum-level bar chart. **F** Genus-level stacked bar chart. **G** Differential taxa identified across groups

To investigate whether BV modulates LPS-induced alterations in pulmonary microbial ecology, lung microbiota profiles were assessed by 16S rRNA sequencing. Analysis of the Shannon and Chao indices indicated that LPS stimulation did not significantly reduce microbial richness or diversity, whereas the BV + LPS group exhibited lower bacterial abundance and evenness (Fig. 3A and B). Venn diagram analysis further revealed a large core microbiota shared across all groups, with the LPS group possessing the highest number of unique taxa, suggesting greater divergence in microbial composition (Fig. 3C). PCA showed treatment-dependent clustering patterns (Fig. 3D).

At the phylum level (Fig. 3E), Bacillota and Bacteroidota dominated across all groups, Pseudomonadota remained relatively stable, and the BV + LPS group had a lower abundance of Actinomycetota than the other two groups.

At the genus level (Fig. 3F), LPS treatment significantly increased the relative abundance of *Faecalibacterium* but decreased that of *Alistipes*, *Bacteroides*, unclassified *Lachnospiraceae*, and unclassified *Lactobacillaceae*, which are considered beneficial functional taxa. BV intervention substantially restored the microbial community structure toward that of the Sal group and restricted the expansion of potentially harmful taxa under inflammatory conditions.

Among significantly different low-abundance genera (Fig. 3G), the LPS group exhibited elevated *Butyricimonas* and reduced *Parasutterella* abundance. BV supplementation significantly increased the abundance of *Flavonifractor*, *Pseudoflavonifractor, Rikenella*, and the *Ruminococcaceae NK4A214 *group. These taxa are associated with short-chain fatty acid (SCFA) production and polyphenol metabolism, indicating that BV reshaped the rare bacterial community structure toward a state similar to the Sal group.

### BV supplementation partially reverses LPS‑induced metabolic dysregulation in broiler lungs

**Fig. 4:**
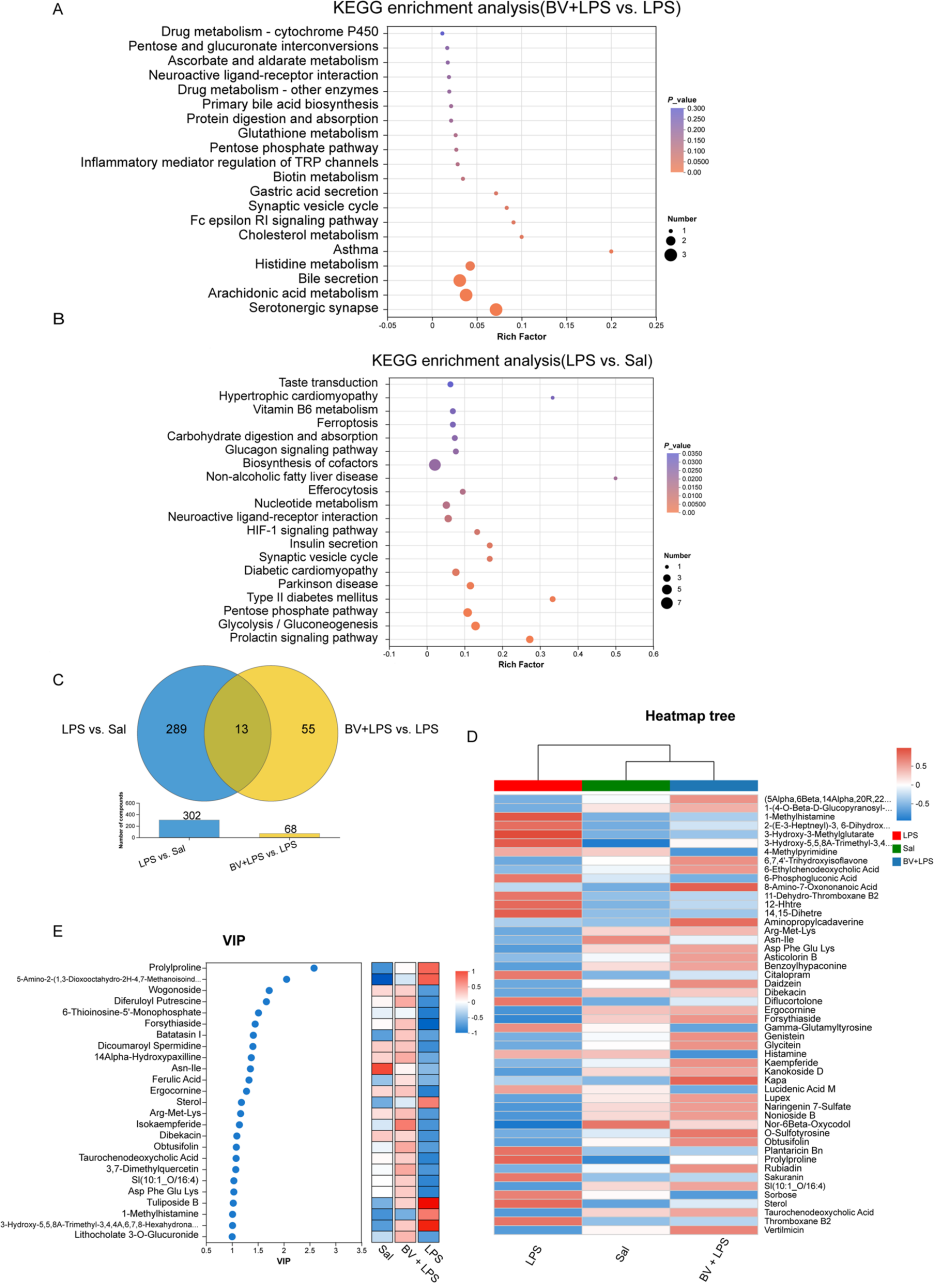
Untargeted metabolomic profiling of broiler lung tissue (*n* = 6). **A** KEGG pathway enrichment analysis comparing BV + LPS vs. LPS. **B** KEGG pathway enrichment analysis comparing LPS vs. Sal. **C** Venn diagram illustrating shared and unique metabolites among treatment groups. **D** Hierarchical clustering analysis of metabolites. **E** VIP score ranking of key metabolites

To test whether metabolic remodeling underlies the protection conferred by BV, untargeted LC–MS metabolomics was performed on lung tissues. LPS exposure caused extensive metabolic reprogramming, as shown by volcano plot analysis (Fig. S2A and B), with 178 metabolites significantly up-regulated and 124 down-regulated. Venn analysis (Fig. 4C) showed markedly fewer differential metabolites in BV + LPS vs. LPS than in LPS vs. Sal, with limited overlap between the two comparisons.

KEGG enrichment analysis (Fig. 4A and B) reflected these shifts, pointing to alterations in energy, lipid, and redox-related pathways, although such enrichment was considered supportive rather than definitive for mechanistic inference.

Hierarchical clustering analysis and variable importance in projection (VIP) ranking (Fig. 4D and E) demonstrated that the metabolic profile of the BV + LPS group closely resembled that of the Sal group, while substantially diverging from the LPS group, underscoring the ability of BV to restore metabolic homeostasis. Potent inflammatory mediators such as histamine and thromboxane B_2_ were markedly elevated following LPS challenge, whereas BV supplementation significantly suppressed their accumulation, restoring levels to near physiological norms. Moreover, BV addition promoted the synthesis of antioxidants, key nutrients, and metabolites essential for maintaining normal cellular functions, supporting its anti-inflammatory and tissue-protective properties.

### BV modulates pulmonary taxa and elevates lung isoflavone levels

**Fig. 5:**
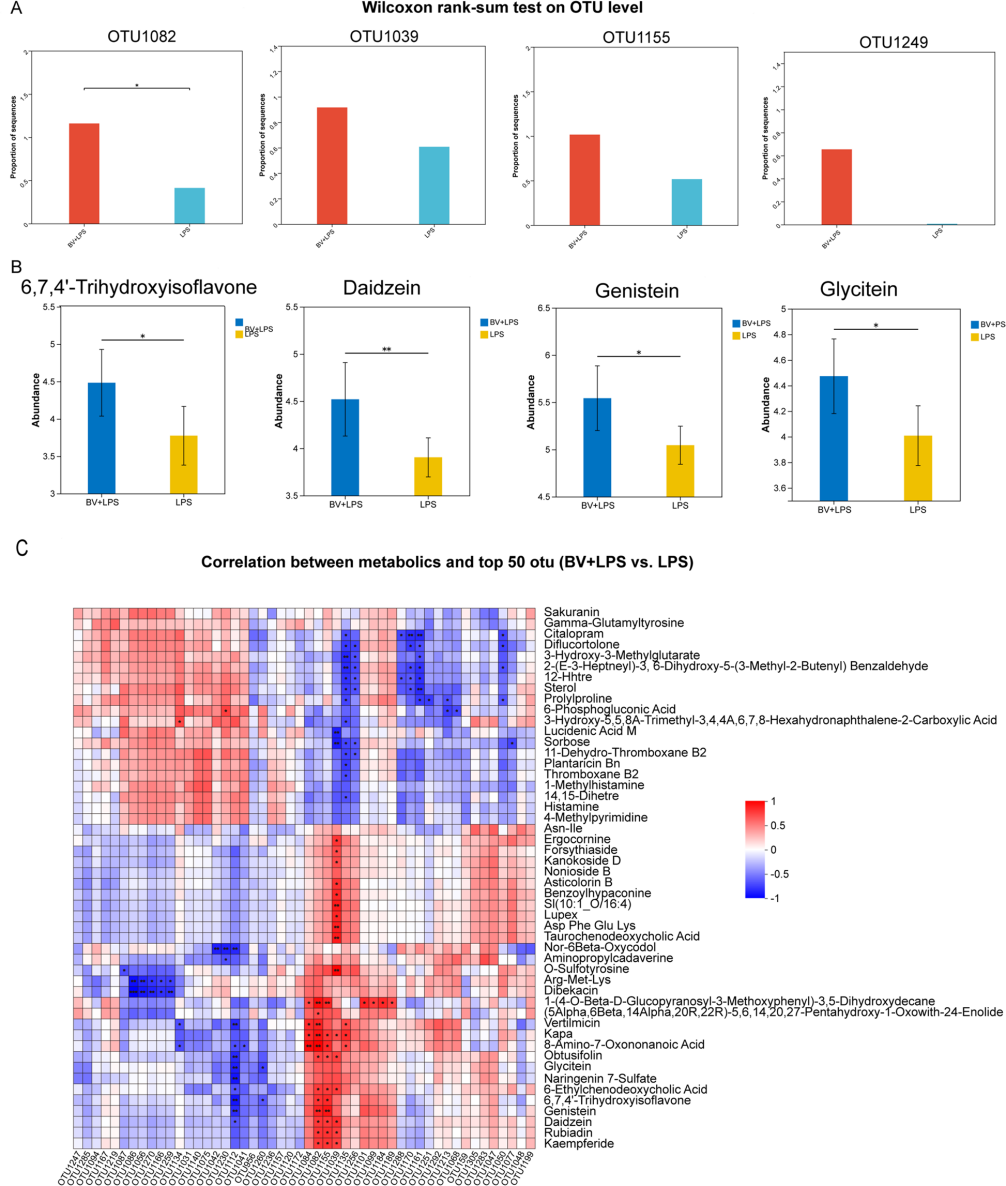
Integrated lung metabolomic and microbiomic analysis comparing BV + LPS vs. LPS treatment. **A** OTU-level differences identified by Wilcoxon rank-sum test (examples: OTU1082 *Blautia*, OTU1039 *Eisenbergiella*, OTU1155 unclassified *Lachnospiraceae*, OTU1249 *Bacteroides*). **B** profiling of isoflavone metabolites. **C** Spearman correlation heatmap between metabolites and the top-50 OTUs (BV + LPS vs. LPS); red and blue denote positive/negative correlations. Data are presented as mean ± SEM. ^*^P < 0.05, ^**^P < 0.01

Integrated lung metabolome-microbiome analysis comparing the BV + LPS group with the LPS group revealed that several BV-enriched OTUs increased in relative abundance, including OTU1082 (*Blautia*), OTU1039 (*Eisenbergiella*), OTU1155 (unclassified *Lachnospiraceae*), and OTU1249 (*Bacteroides*) (Fig. 5A). Concurrently, pulmonary concentrations of the isoflavones daidzein, genistein, and glycitein, as well as the downstream metabolite 6,7,4'-trihydroxyisoflavone, were higher in BV + LPS group than in LPS group (Fig. 5B), while levels of the potent inflammatory mediators thromboxane B_2_ and histamine were significantly reduced (Fig. S2C and D).

Correlation analysis demonstrated that the relative abundances of OTU1082 (*Blautia*) and OTU1155 (unclassified *Lachnospiraceae*) were positively and significantly associated with concentrations of the aforementioned isoflavones (Fig. 5C).

These patterns suggest a potential link between BV-driven shifts in specific bacterial members and enhanced isoflavone abundance, alongside a reduction in inflammatory metabolite signals.

### LPS alters immune and defense-associated pathways in the lung, while BV modulates lipid metabolism and matrix-interaction signaling

**Fig. 6:**
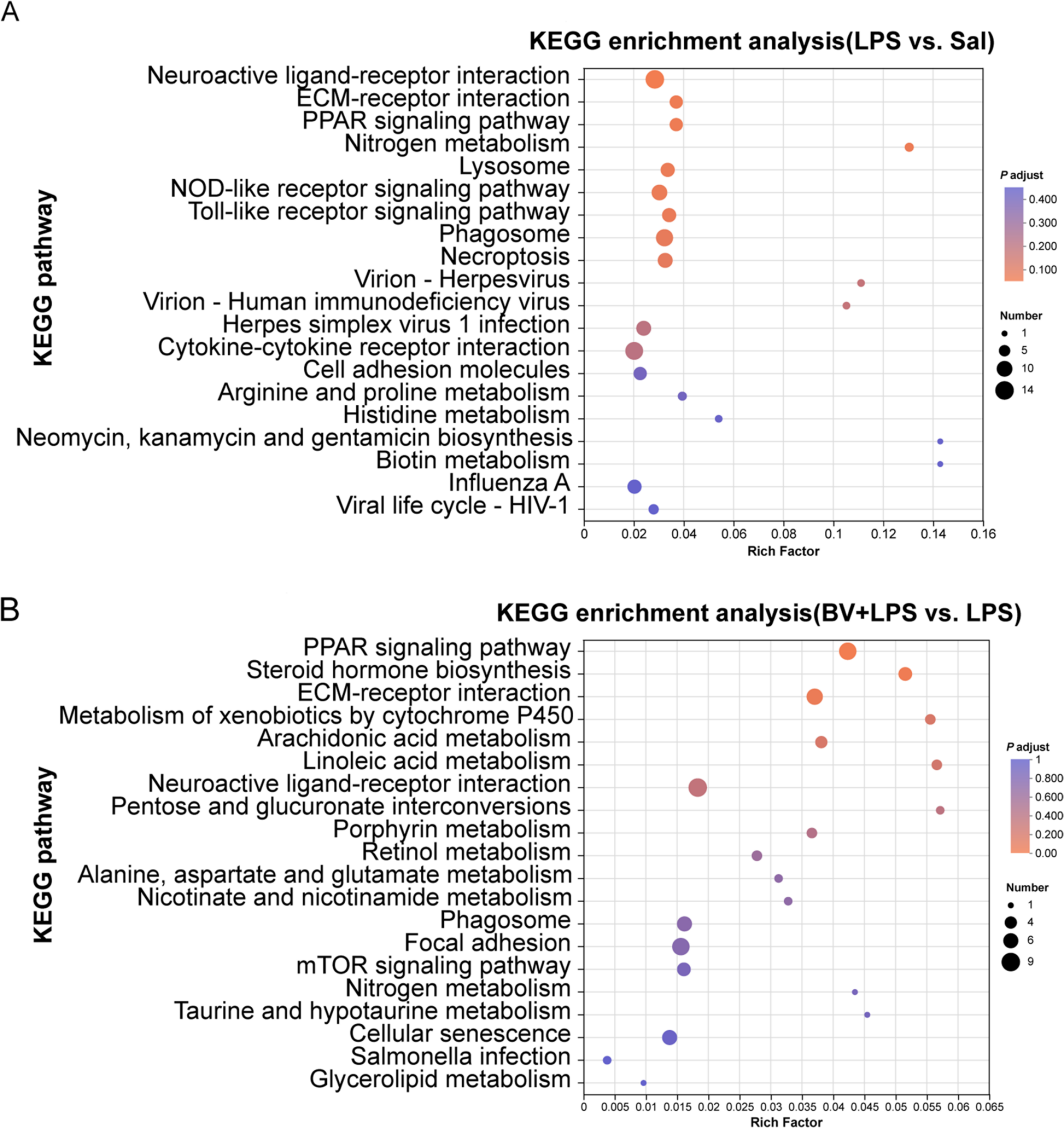
KEGG pathway enrichment of DEGs in broiler lungs (*n* = 3). **A** LPS vs. Sal. **B** BV + LPS vs. LPS. Bubble size indicates the number of DEGs per pathway; color denotes adjusted* P* value (*P* adjust); rich factor represents the ratio of DEG hits to pathway size

Transcriptomic analysis revealed 91 up-regulated and 214 down-regulated genes in the LPS group compared with the Sal group (Fig. S3A and B). GO analysis showed that, following LPS exposure, differentially expressed genes (DEGs) were enriched in 477 GO terms, with significant enrichment in bacterial defense responses, immune system processes, immune response pathways, and ribosome-related processes (Fig. S3C). KEGG enrichment of these DEGs indicated predominant enrichment in extracellular matrix (ECM)-receptor interaction, NOD-like receptor signaling, Toll-like receptor signaling, and phagosome pathways (Fig. 6A), reflecting inflammatory activation and tissue microenvironment remodeling.

In contrast, DEGs in the BV + LPS group relative to the LPS group (120 up-regulated, 101 down-regulated) showed GO term enrichment in extracellular region organization, collagen trimer formation, fatty acid binding, enzyme inhibitor activity, and arachidonic acid cyclization processes (Fig. S3D). KEGG analysis revealed prominent enrichment in peroxisome proliferator-activated receptor (PPAR) signaling, steroid biosynthesis, ECM-receptor interaction, and arachidonic acid metabolism pathways (Fig. 6B). These enrichment patterns suggest that BV shifts the LPS-driven transcriptional response from an inflammation and phagosome-centered program toward one characterized by lipid receptor signaling, detoxification, and matrix adhesion/repair, indicating a transition toward an anti-inflammatory and tissue-repair phenotype at the molecular level.

### Elevated isoflavone levels in the BV + LPS group were associated with increased PPAR-γ signaling, reduced NF‑κB activity, and attenuated inflammatory responses

**Fig. 7:**
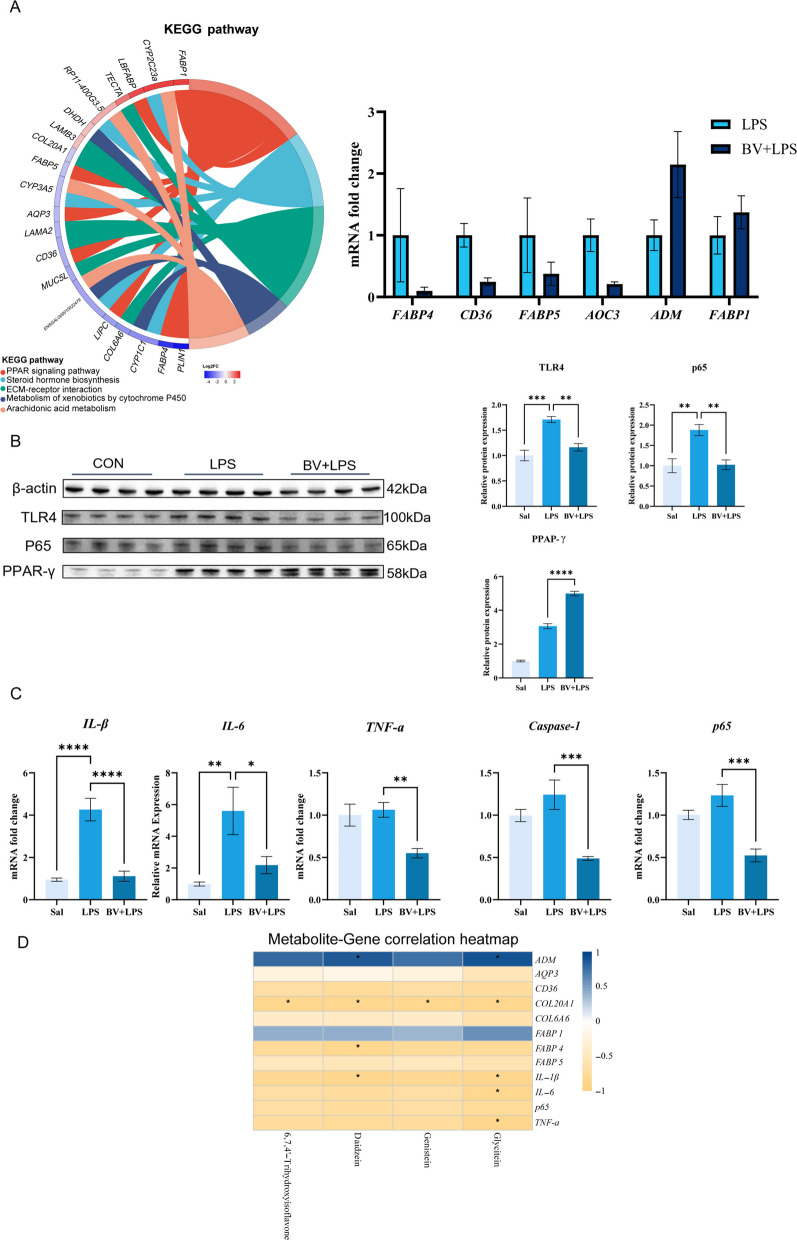
Integrated analysis of transcriptomic data, metabolite profiles, and molecular assays in broiler lung tissue, **A** enrichment chord plot from transcriptome analysis and RT-qPCR validation. **B** WB quantification of PPAR-γ, TLR4, and p65 protein expression (*n* = 4). **C** RT-qPCR measurement of *IL‑1β*, *IL‑6*, *TNF‑α*, *caspase-1*, and *p65* transcriptional levels (*n* = 5). **D** correlations between representative isoflavone metabolites and PPAR pathway, NF‑κB pathway genes, and pro‑inflammatory cytokines. Data are presented as mean ± SEM. ^*^P < 0.05, ^**^P < 0.01

Further validation of transcriptomic findings indicated that BV supplementation reshaped lipid-sensing and inflammatory signaling in LPS-exposed lungs. Within the PPAR signaling pathway, the expression of genes such as *FABP1* was increased, while the expression of *FABP4*, *FABP5*, and *CD36* were reduced in the BV + LPS group compared to the LPS group, trends that were confirmed by RT-qPCR (Fig. 7A).

WB analysis further demonstrated higher PPAR-γ protein and lower TLR4 and NF-κB p65 in the BV + LPS group compared with LPS group, while LPS alone elevated TLR4 and p65 relative to saline (*P* < 0.05; Fig. 7B).

Consistently, RT-qPCR revealed that transcriptional levels of the pro-inflammatory cytokines *IL‑1β* and *IL‑6* were significantly elevated in the LPS group. BV + LPS treatment significantly reduced mRNA expression of *IL‑1β*, *IL‑6*, *TNF‑α*, *caspase-1*, and *p65* (*P* < 0.05; Fig. 7C). Correlation analysis linking pulmonary metabolites to gene expression showed that isoflavone metabolites were positively associated with PPAR‑axis genes and negatively associated with *p65* and pro‑inflammatory genes (Fig. 7D). These data support a model linking BV-associated isoflavones to PPAR-γ activation and NF-κB-driven inflammation attenuation.

### Genistein and daidzein attenuate LPS-induced inflammation via PPAR-γ signaling in HD11 cells

**Fig. 8:**
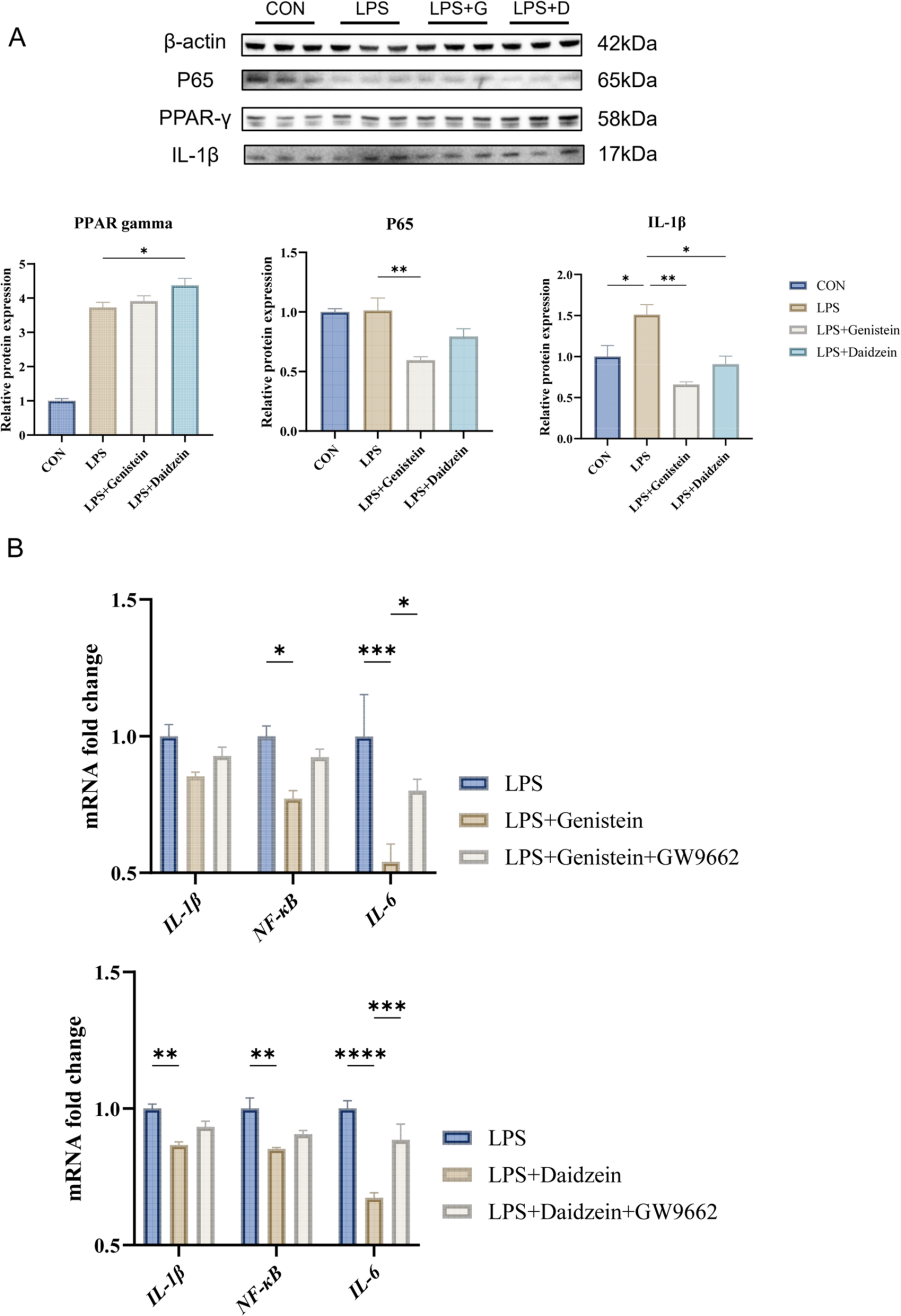
Effects of genistein, daidzein and GW9662 on inflammatory responses in LPS-stimulated HD11 cells. **A** Western blot analysis of p65, PPAR-γ, and IL-1β protein expression in three groups (*n* = 3). **B** RT-qPCR analysis of *IL-1β*, *NF-κB*, and *IL-6* mRNA expression in three groups (*n* = 3). Data are presented as mean ± SEM. ^*^P < 0.05, ^**^P < 0.01, ^***^P < 0.001, ^****^P < 0.0001

Isoflavone intervention further supported the involvement of PPAR-γ signaling in HD11 cells. Western blot analysis showed that both genistein and daidzein increased PPAR-γ protein expression while decreasing p65 and IL-1β levels under LPS stimulation (Fig. 8A). Notably, daidzein showed a slightly stronger inhibitory effect than genistein.

To determine whether these effects were PPAR-γ-dependent, we performed a pharmacological inhibition assay using GW9662. The addition of GW9662 partially abolished the protective effects of isoflavones, specifically by increasing *p65*,* IL-1β* and *IL-6* expression (Fig. 8B). These findings indicate that genistein and daidzein mitigate LPS-induced inflammatory injury in HD11 cells, at least in part, through a PPAR-γ-dependent mechanism.

### BV supernatant shows anti-inflammatory effects in macrophages

**Fig. 9:**
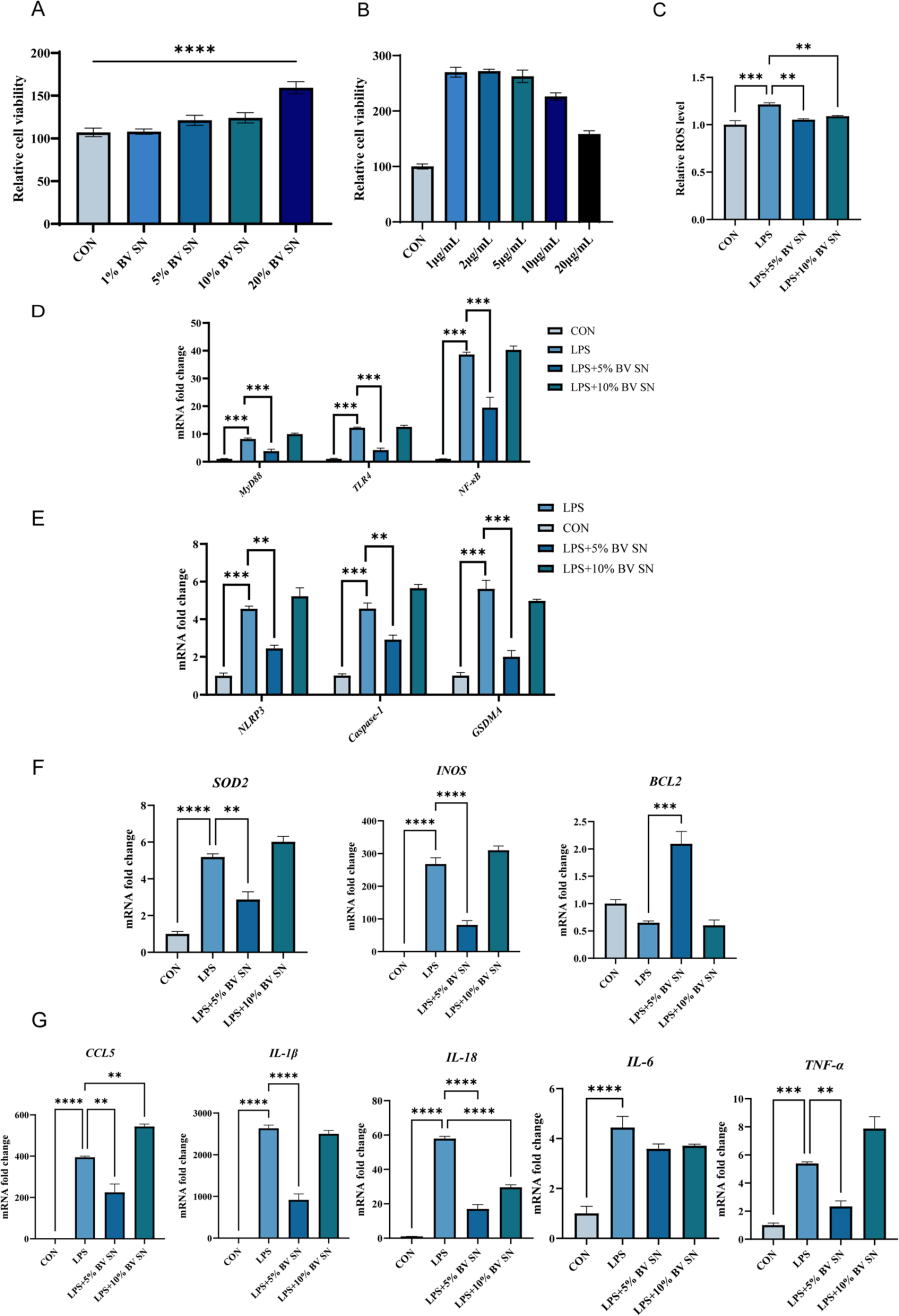
Effects of BV supernatant on LPS‑induced oxidative stress and inflammatory gene expression in HD11 cells. **A** and **B** Assessment of HD11 cell viability (*n* = 5). **C** Intracellular ROS levels (*n* = 4). **D** NF-κB-axis genes (*TLR4*, *MyD88*, *p65*) (*n* = 3). **E** inflammasome/pyroptosis-related genes (*NLRP3*, *caspase-1*, *GSDMA*) (*n* = 3). **F** oxidative stress/anti-apoptotic markers (*SOD2*, *iNOS*, *BCL2*) (*n* = 3). **G** chemokines/cytokines (*CCL5*, *IL-1β*, *IL-6*, *TNF-α*) (*n* = 3). Data are presented as mean ± SEM. ^*^P < 0.05, ^**^P < 0.01, ^***^P < 0.001

We used HD11 macrophages to evaluate whether BV culture supernatant (BV SN) directly protects against LPS-induced oxidative stress and inflammatory responses. CCK-8 screening confirmed that BV SN at 5% and 10% (v/v) did not compromise cell viability after 6 h and was therefore selected for subsequent experiments, whereas LPS was applied at 1 μg/mL (Fig. 9A and B). Under these conditions, LPS markedly increased intracellular ROS accumulation, which was significantly attenuated by BV SN at both doses (*P* < 0.05; Fig. 9C).

At the transcriptional level, LPS induced robust activation of the NF-κB signaling cascade, as evidenced by the up-regulation of *TLR4*, *MyD88*, and *p65*, together with elevated expression of inflammasome/pyroptosis-associated genes (*NLRP3*, *caspase-1*, and *GSDMA*). Concurrent increases in oxidative stress-related markers (*SOD2* and *iNOS*) and a reduction in the anti-apoptotic gene *BCL2* were also observed, alongside pronounced induction of the chemokine *CCL5* and multiple pro-inflammatory cytokines (*IL-1β*, *IL-6*, and *TNF-α*) (*P* < 0.05; Fig. 9D–G). BV SN treatment largely reversed these transcriptional alterations, suppressing *TLR4/MyD88/p65* and *NLRP3/caspase-1/GSDMA* expression, reducing *SOD2* and *iNOS*, restoring *BCL2*, and down-regulating *CCL5* and inflammatory cytokines toward control levels. Both 5% and 10% BV SN exhibited comparable efficacy (*P* < 0.05), consistent with the anti-inflammatory patterns observed in vivo.

### BV supernatant dampens NF-κB/NLRP3 signaling and shifts macrophage phenotype toward a resolution profile in LPS-stimulated HD11 cells

**Fig. 10:**
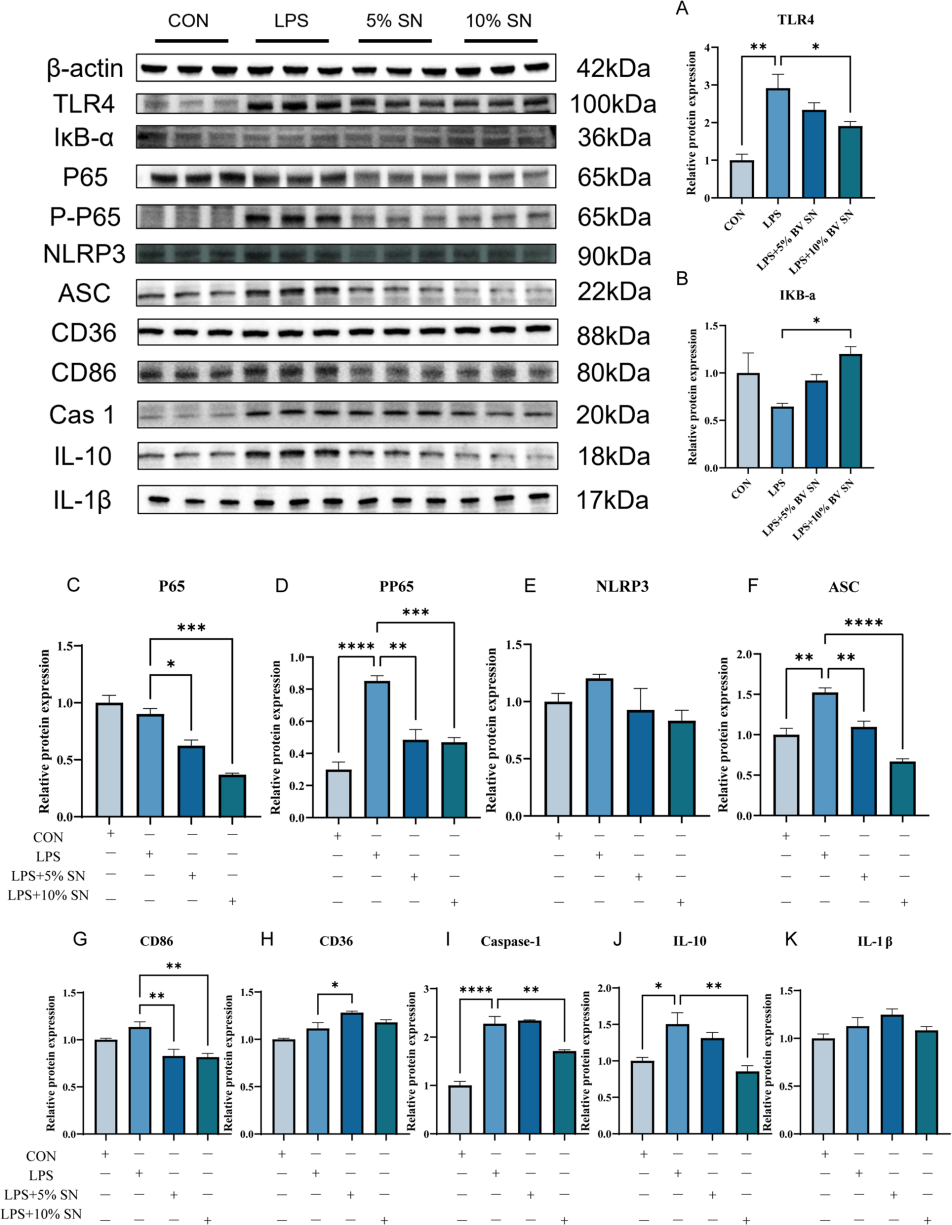
Western blot analysis of LPS‑induced inflammatory signaling protein expression and macrophage polarization markers in HD11 cells (*n* = 3). **A** TLR4. **B** IκB‑α. **C** p65. **D** p‑p65. **E** NLRP3. **F** ASC. **G** CD86. **H** CD36. **I** caspase‑1. **J** IL‑10. **K** IL‑1β. Data are presented as mean ± SEM. ^*^P < 0.05, ^**^P < 0.01, ^***^P < 0.001

Compared with the control group, LPS stimulation markedly increased expression of TLR4, phosphorylated p65 (p-p65), and ASC (*P* < 0.05; Fig. 10A, D, and F), while significantly reducing IκB-α levels (Fig. 10B). Treatment with BV SN significantly suppressed LPS-induced TLR4 expression, restored IκB-α protein levels, reduced NLRP3 abundance, and markedly lowered p65, p-p65, and ASC expression (*P* < 0.05), with 10% BV SN exerting a more pronounced inhibitory effect.

BV SN also significantly down-regulated CD86, a marker of M1 macrophage polarization, in LPS-treated cells (*P* < 0.05; Fig. 10G), while up-regulating the scavenger receptor CD36 (Fig. 10H). Furthermore, 10% BV SN markedly suppressed caspase-1 and IL-10 expression (*P* < 0.05; Fig. 10I and J), although no significant changes were observed in intracellular IL-1β protein levels (Fig. 10K).

## Discussion

In intensive farming environments, the concentration of microbial aerosols in livestock houses is significantly higher than in the external atmosphere. LPS, a key component of Gram-negative bacterial cell walls, activates the host innate immune system and triggers strong inflammatory responses, leading to airway inflammation, oxidative stress, and decreased growth performance in broilers [18–21]. This study established a broiler model of chronic LPS intratracheal instillation to systematically evaluate the mitigating effects of BV and, through integrated multi-omics and in vitro validation experiments, to elucidate the multi-pathway mechanisms by which BV alleviates LPS-induced chronic pneumonia.

This study confirmed that chronic LPS exposure significantly reduced live body weight and slaughter weight of broilers and induced typical lung histopathological damage, such as thickened alveolar septa, dilated alveolar spaces, and erythrocyte infiltration. Concurrently, levels of pro-inflammatory cytokines (IL-1β, IL-6, TNF-α) in serum and BALF increased, the anti-inflammatory mediator IL-10 was suppressed, and antioxidant capacity was imbalanced (e.g., MDA increased, while T-AOC, SOD, and GSH-Px were compensated by elevation but CAT activity decreased), indicating significant activation of systemic inflammation and oxidative stress. BV supplementation effectively reversed these phenotypes, restored growth performance, alleviated tissue damage, and regulated inflammatory and oxidative indicators toward normal levels, confirming its mitigating effect on chronic respiratory inflammation.

Recent research has confirmed that the lungs are not sterile; although the density of the lung microbiota is low, it plays a crucial role in local immune regulation and barrier maintenance [22–25]. In this study, although LPS stimulation did not significantly alter the overall richness or Shannon diversity of the lung microbiota, it caused significant structural reconstruction at the genus level, manifested by decreased relative abundances of potentially metabolically and immunomodulatory functional groups such as *Alistipes*, *Bacteroides*, unclassified *Lachnospiraceae*, and unclassified *Lactobacillaceae*, while the relative abundance of *Faecalibacterium* increased. These changing taxa are closely related to SCFA and polyphenol metabolism. For instance, *Alistipes* can induce the anti-inflammatory cytokine IL-10 via TLR2 signaling, alleviating intestinal inflammation [26]; *Bacteroides*-produced polysaccharide A possesses anti-inflammatory and immunomodulatory functions [27], and members of Lactobacillaceae can modulate the immune system and enhance mucosal barrier function [28]. Therefore, LPS might disrupt the pulmonary microecological balance by selectively suppressing these functional taxa. BV intervention significantly restored the structure of the microbiota and further promoted the proliferation of genera associated with SCFA and polyphenol metabolism, such as *Flavonifractor*, *Rikenella*, and *Ruminococcaceae_NK4A214_group*, while also increasing the relative abundance of genera like *Blautia* and *Eisenbergiella*, creating a foundation for systemic anti-inflammatory effects at the microecological level.

Regarding the mechanism underlying these pulmonary microbial alterations, our data suggest a predominantly local, transient modulatory effect rather than direct BV colonization. Notably, 16S rRNA profiling revealed no significant enrichment of *Bacillus* in lung tissue following BV administration. Given the direct intrapulmonary delivery route employed in this study, BV and its bioactive metabolites likely exerted immediate effects on the local lung microenvironment, thereby reshaping resident microbial composition without establishing persistent colonization. This interpretation is supported by previous evidence showing that even non-colonizing microorganisms can regulate surrounding microbial communities through quorum-sensing molecules and secondary metabolites [29], while metabolites such as SCFA and succinate can modulate pathogen metabolism and virulence [30]. Nevertheless, the precise molecular basis of BV-driven community remodeling requires further dedicated research.

Integrated metabolomic and microbiomic analysis further revealed that key BV-enriched taxa (e.g., OTU1082 *Blautia* and OTU1155 unclassified *Lachnospiraceae*) possess significant isoflavone transformation capabilities. Previous studies have reported that *Blautia* can deglycosylate flavonoid glycosides to generate active aglycones [31], and Lachnospiraceae members also possess β-glucosidase activity, converting genistin and daidzin into their active forms, genistein and daidzein [32]. Data from this study showed significantly increased levels of daidzein, genistein, glycitein, and their downstream metabolite 6,7,4′-trihydroxyisoflavone in the lung tissues of the BV + LPS group, which were positively correlated with the abundance of the aforementioned microbiota. Isoflavones play an important role in the intestine [33, 34]. Simultaneously, levels of potent pro-inflammatory mediators such as thromboxane B_2_ and histamine were significantly decreased, which have core pro-inflammatory functions in tissues [35–38]. These results suggest that BV may enhance the local levels of active isoflavones by modulating the lung microbiota and their isoflavone conversion capacity, while inhibiting the accumulation of key pro-inflammatory mediators.

Global metabolomic analysis indicated that LPS induced extensive metabolic reprogramming in lung tissue. KEGG enrichment analysis suggested the activation of typical inflammation-related metabolic pathways such as glycolysis/gluconeogenesis, pentose phosphate pathway, and HIF-1 signaling pathway. As a central transcription factor, hypoxia‑inducible factor‑1α not only upregulates glycolytic enzymes [39] but also drives immune cells toward a highly glycolytic, pro‑inflammatory phenotype [40–42]. In parallel, the pentose phosphate pathway generates NADPH to counteract LPS‑induced ROS [39]. BV intervention restored the overall metabolic profile toward a healthy state and specifically enriched anti-inflammatory and antioxidant pathways such as arachidonic acid metabolism and glutathione metabolism, supporting its tissue-protective effects at the metabolic level.

Transcriptomic analysis provided direct evidence for the above mechanisms at the gene expression level. LPS activated the TLR and NOD-like receptor signaling pathways and the phagosome pathway, which can promote NF‑κB activation and NLRP3 inflammasome assembly, leading to the maturation of pro‑inflammatory cytokines and pyroptotic cell death, thereby amplifying inflammatory injury [43–46]. In this study, the significant upregulation of related genes in the LPS group confirmed the strong activation of these pathways. Further analysis revealed that BV intervention significantly suppressed these inflammatory signals and instead enriched pathways such as PPAR signaling, steroid biosynthesis, and ECM-receptor interaction. In the BV + LPS group, PPAR-γ protein expression was significantly upregulated in lung tissue, while TLR4 and NF-κB p65 protein levels decreased. RT-qPCR results further showed increased expression of the PPAR pathway-related gene *FABP1*, and decreased expression of *FABP4*, *FABP5*, and *CD36*. Correlation analysis clearly showed that isoflavone metabolites were positively correlated with PPAR pathway genes (*FABP1*, *CD36*) and negatively correlated with the NF-κB pathway gene (*p65*) and pro-inflammatory factors (*IL-1β*, *IL-6*, *TNF-α*, *caspase 1*), thereby revealing a "microbiota–isoflavone–PPARγ–NF-κB" regulatory pathway through which BV alleviates pulmonary inflammation at the molecular level.

We further confirmed in vitro that isoflavones (genistein and daidzein) attenuate LPS-induced inflammatory injury in HD11 macrophages, which was accompanied by increased PPAR-γ and reduced p65/IL-1β signaling. In line with these results, previous studies have reported that genistein and daidzein activate PPAR-γ and suppress NF-κB transcriptional activity in macrophages [38, 47, 48]. To further verify pathway dependence, GW9662 inhibition assays were performed, and GW9662 partially reversed the isoflavone-mediated effects. These findings support a PPAR-γ-dependent mechanism for isoflavone-mediated protection against LPS-induced inflammation.

As complementary evidence, BV culture supernatant also produced direct anti-inflammatory and antioxidative effects in HD11 cells, including suppression of TLR4/NF-κB/NLRP3-related signaling, reduced ROS accumulation, and a shift toward a less pro-inflammatory phenotype, broadly consistent with the in vivo observations.

This study has several limitations. First, BV was administered by intratracheal instillation to ensure precise pulmonary delivery and to reduce intestinal interference. This route is suitable for mechanism-focused research, but it differs from routine field application methods such as spray or oral delivery. Second, although BV clearly altered pulmonary microbial structure, the exact mechanism of this remodeling remains incompletely defined. Our data support a local and transient modulatory effect rather than stable BV colonization, but the precise molecular processes by which BV and its metabolites reshape lung microbial communities still need further investigation. Finally, the in vitro experiments were performed in HD11 cells. Although this model is widely used in avian immunology, it cannot fully capture the complexity of the in vivo lung microenvironment.

## Conclusion

This study systematically elucidated the molecular mechanism by which *Bacillus velezensis* alleviates LPS-induced chronic pneumonia in broilers through a core "microbiota–isoflavone–PPAR-γ–NF-κB" regulatory axis. BV remodeled the pulmonary microbiota, enhanced isoflavone bioconversion capacity, and increased the levels of bioactive isoflavones in lung tissue, thereby activating PPAR-γ signaling and suppressing NF-κB activation and downstream NLRP3 inflammasome signaling. Importantly, in vitro experiments further confirmed that representative isoflavones (genistein and daidzein) attenuated LPS-induced inflammatory injury, and GW9662 partially reversed these effects, supporting a PPAR-γ-dependent mechanism. In addition, BV-derived metabolites directly acted on macrophages, promoted a shift toward an anti-inflammatory/repair phenotype, reduced pro-inflammatory mediator release, and alleviated oxidative stress in lung tissue and at the systemic level, thereby facilitating tissue repair. Future studies should identify the core anti-inflammatory components in BV supernatant and further define their mechanisms of action to support probiotic-based strategies for improving animal and human health.

## Supplementary Information

Additional file 1: Table S1. Primer sequences used for RT-qPCR analysis. Table S2. Detailed information of the primary and secondary antibodies. Table S3. Hematological parameters of experimental subjects (*n* = 7). Fig. S1. Effects of BV supplementation on inflammatory cytokine levels in broiler serum and BALF (*n* = 6). (A) Levels of inflammatory cytokines in broiler serum. (B) Levels of inflammatory cytokines in broiler BALF. Fig. S2. Metabolic reprogramming in broiler lungs following different treatments (*n* = 6). Volcano plots identifying differentially abundant metabolites (DAMs) between (A) BV + LPS and LPS groups; (B) LPS and Saline groups; (C) Levels of histamine; (D) thromboxane B_2_. Fig. S3. Transcriptomic analysis of broiler lungs. (A) Volcano plot of DEGs in the BV + LPS group vs. the LPS group (*n* = 3). (B) Volcano plot of DEGs in the LPS group vs. the Sal group. (C) GO enrichment analysis of DEGs from LPS vs. Saline comparison. (D) GO enrichment analysis of DEGs from the BV + LPS vs. LPS comparison. Fig. S4 Uncropped PVDF membranes for Western blot analysis of broiler lung tissues and HD11 cells. Fig. S5. Uncropped PVDF membranes for Western blot analysis of isoflavone-treated HD11 cells showing protein marker transfer pattern.

## Data Availability

All data that support the results of this study are available from the corresponding author on reasonable request.

## Abbreviations

ASC: Apoptosis-associated speck-like protein containing a CARD
BV: *Bacillus velezensis*
CCK-8: Cell Counting Kit-8
DEGs: Differentially expressed genes
GSDMA: Gasdermin A
KEGG: Kyoto Encyclopedia of Genes and Genomes
MyD88: Myeloid differentiation primary response 88
NF-κB: Nuclear factor kappa-light-chain-enhancer of activated B cells
NLRP3: NLR family pyrin domain containing 3
OTUs: Operational taxonomic units
PPAR: Peroxisome proliferator-activated receptor
qRT-PCR: Quantitative real-time polymerase chain reaction
RIPA: Radio immunoprecipitation assay
T-AOC: Total antioxidant capacity
TLR4: Toll-like receptor 4
VIP: Variable importance in projection
