# Supplementary material for: Bacillus velezensis mitigates chronic LPS-induced lung injury of broilers via microbiota-driven isoflavone production and NF-κB/PPAR-γ axis modulation
Source: J Anim Sci Biotechnol. 2026 May 7;17:85. doi: 10.1186/s40104-026-01391-1 (PMC13151109; doi:10.1186/s40104-026-01391-1)
Supplement: Supplementary file 1 — Additional file 1: Table S1. Primer sequences used for RT-qPCR analysis. Table S2. Detailed information of the primary and secondary antibodies. Table S3. Hematological parameters of experimental subjects (n = 7). Fig. S1. Effects of BV supplementation on inflammatory cytokine levels in broiler serum and BALF (n = 6). (A) Levels of inflammatory cytokines in broiler serum. (B) Levels of inflammatory cytokines in broiler BALF. Fig. S2. Metabolic reprogramming in broiler lungs following different treatments (n = 6). Volcano plots identifying differentially abundant metabolites (DAMs) between (A) BV + LPS and LPS groups; (B) LPS and Saline groups; (C) Levels of histamine; (D) thromboxane B2. Fig. S3. Transcriptomic analysis of broiler lungs. (A) Volcano plot of DEGs in the BV + LPS group vs. the LPS group (n = 3). (B) Volcano plot of DEGs in the LPS group vs. the Sal group. (C) GO enrichment analysis of DEGs from LPS vs. Saline comparison. (D) GO enrichment analysis of DEGs from the BV + LPS vs. LPS comparison. Fig. S4 Uncropped PVDF membranes for Western blot analysis of broiler lung tissues and HD11 cells. Fig. S5. Uncropped PVDF membranes for Western blot analysis of isoflavone-treated HD11 cells showing protein marker transfer pattern. [file 40104_2026_1391_MOESM1_ESM.doc]

**Table S1** Primer sequences used for RT-qPCR analysis.

| Gene name | Primer sequence(5'-3') |
| --- | --- |
| *β-actin* | F:ACCTGAGCGCAAGTACTCTGTCT |
|  | R:CATCGTACTCCTGCTTGCTGAT |
| *IL-1β* | F:CAGCCTCAGCGAAGAGACCTT |
|  | R:ACTGTGGTGTGCTCAGAATCC |
| *IL-6* | F:TTCACCGTGTGCGAGAACAGC |
|  | R:CAGCCGTCCTCCTCCGTCAC |
| *IL-18* | F:TGATGAGCTGGAATGCGATG |
|  | R:ACTGCCAGATTTCACCTCCTG |
| *CCL5* | F:TCTTCATCTCCGTTTGGGGC |
|  | R:GACTTTGCTGCATGGACTCG |
| *iNOS* | F:GAAGTGGTATGCTCTGCCTGCTG |
|  | R:GTCTCGCACTCCAATCTCTGTTCC |
| *TLR4* | F:GAATGACACGGACACTCTT |
|  | R:ACATAGGAACCTCTGACAAC |
| *NF-κB p65* | F:CACATGGTGGTGACCGCCAATAG |
|  | R:GTGCCATCGTATGTAGTGCTGTCC |
| *caspase-3* | F:AGCAGACAGTGGACCAGATGAAAC |
|  | R:GGCGTGTTCCTTCAGCATCCTAC |
| *caspase-1* | F:GTGCTGCCGTGGAGACAACATAG |
|  | R:AGGAGACAGTATCAGGCGTGGAAG |
| *GSDMA* | F:TGCTGCTATTGCTGGAGTCTTTGG |
|  | R:CTGCTGCTGCTCGCTGAAGG |
| *FABP5* | F:TGGCCATCGACGCGTTTTTA |
|  | R:TCTGGTTTCGCCATGCTTCC |
| *FABP1* | F:TCTGAAAGCTCTTGCACTGCC |
|  | R:TCACAGTCTGCCTGGGTGTT |
| *ADM* | F: GCACGTTGTTTACTTCACGCA |
|  | R: GCACAGTTTCCCGAGGAG |
| *CD36* | F: AGTCTTCTACCCGTATAATGGGA |
|  | R: CCTTCACGGTCTTACTGGTC |
| *FABP4* | F: GCACCTGGAAGCTCCTTTCT |
|  | R: ATTAGGCTTGGCCACACCAG |
| *AOC3* | F:GGCCTGGATAACTGCTGGTT |
|  | R:GCTGGTGAAAAACACGCCAT |
| *TNF-α* | F: TGATCGTGACACGTCTCTGC |
|  | R: CAACCAGCTATGCACCCCAG |
| *BcL-2* | F: GTCGGAAGCGATCTGCCTTT |
|  | R: GCACCGACAGCTGCATTTAC |
| *MyD88* | F: AAGGTGTCGGAGGATGGTGGTC |
|  | R: GGAATCAGCCGCTTGAGACGAG |
| *SOD2* | F: GACCTGCCCTACGACTATGG |
|  | R: TTGCCAGCGCCTCTTTGTAT |
| *NLRP3* | F: GCTCCTTGCGTGCTCTAAGACC |
|  | R: TTGTGCTTCCAGATGCCGTCAG |

**Table S2** Detailed information of the primary and secondary antibodies.

| Protein | Brand | Catalog Number | Dilution Ratio | Source |
| --- | --- | --- | --- | --- |
| ASC | Immunoway | YM8352 | 1:2000 | rabbit |
| IL-10 | BIOSS | bs-0698R | 1:500 | rabbit |
| caspase-1 | Proteintech | 22915-1-AP | 1:2000 | rabbit |
| TLR4 | Proteintech | 19811-1-AP | 1:1000 | rabbit |
| IκBα | Proteintech | 10268-1-AP | 1:1000 | rabbit |
| NLRP3 | Proteintech | 68102-1-Ig | 1:2000 | mouse |
| IL-1β | Proteintech | 26048-1-AP | 1:1000 | rabbit |
| p-p65 | Proteintech | 82335-1-RR | 1:2000 | rabbit |
| p65 | Proteintech | 10745-1-AP | 1:1000 | rabbit |
| CD36 | Proteintech | 18836-1-AP | 1:500 | rabbit |
| CD86 | BIOSS | bs-43589R | 1:500 | rabbit |
| PPAR-γ | BIOSS | bs-0530R | 1:500 | rabbit |
| β-actin | Proteintech | 66009-1-Ig | 1:1000 | mouse |

**Table S3** Hematological parameters of experimental subjects (n = 7).

| Items |  |  |  |  |  |
| --- | --- | --- | --- | --- | --- |
|  | Sal | LPS | BV + LPS | SEM | *P-*value |
| WBC (10^9^/L) | 67.26±1.81 | 64.56±1.35 | 61.57±2.14 | 1.113 | 0.110 |
| LYM (10^9^/L) | 52.23±1.76 | 50.87±2.04 | 48.34±2.15 | 1.148 | 0.394 |
| MID (10^9^/L) | 5.94±0.32 | 5.70±0.28 | 5.41±0.35 | 0.180 | 0.511 |
| Gran (10^9^/L) | 9.09±1.08 | 7.99±0.95 | 7.81±0.60 | 0.510 | 0.567 |
| RBC (10^12^/L) | 2.35±0.07 | 2.36±0.06 | 2.33±0.07 | 0.037 | 0.939 |
| HGB (g/L) | 130.43±3.07 | 130.00±2.25 | 126.71±3.12 | 1.600 | 0.608 |
| HCT (%) | 29.23±0.88 | 29.20±0.70 | 28.33±1.05 | 0.494 | 0.720 |
| MCV (fL) | 124.63±1.35 | 123.94±1.04 | 121.81±1.11 | 0.697 | 0.237 |
| MCH (pg) | 55.57±1.00 | 55.17±1.20 | 54.54±1.21 | 0.633 | 0.816 |
| MCHC (g/L) | 446.29±5.52 | 445.57±7.67 | 448.43±10.10 | 4.383 | 0.966 |
| RDW-CV (%) | 10.69±0.16 | 10.81±0.15 | 10.53±0.10 | 0.081 | 0.372 |
| PLT (10^9^/L) | 44.14±1.83 | 50.57±2.17 | 47.00±3.73 | 1.594 | 0.268 |
| MPV(fL) | 10.86±0.19 | 11.11±0.32 | 10.99±0.18 | 0.131 | 0.746 |
| PDW (%) | 14.39±0.54 | 14.09±0.47 | 14.64±0.98 | 0.386 | 0.854 |
| PCT (%) | 0.04±0.00 | 0.05±0.00 | 0.05±0.00 | 0.002 | 0.086 |
| P-LCR (%) | 2.73±0.20 | 2.9±0.26 | 3.07±0.75 | 0.262 | 0.879 |


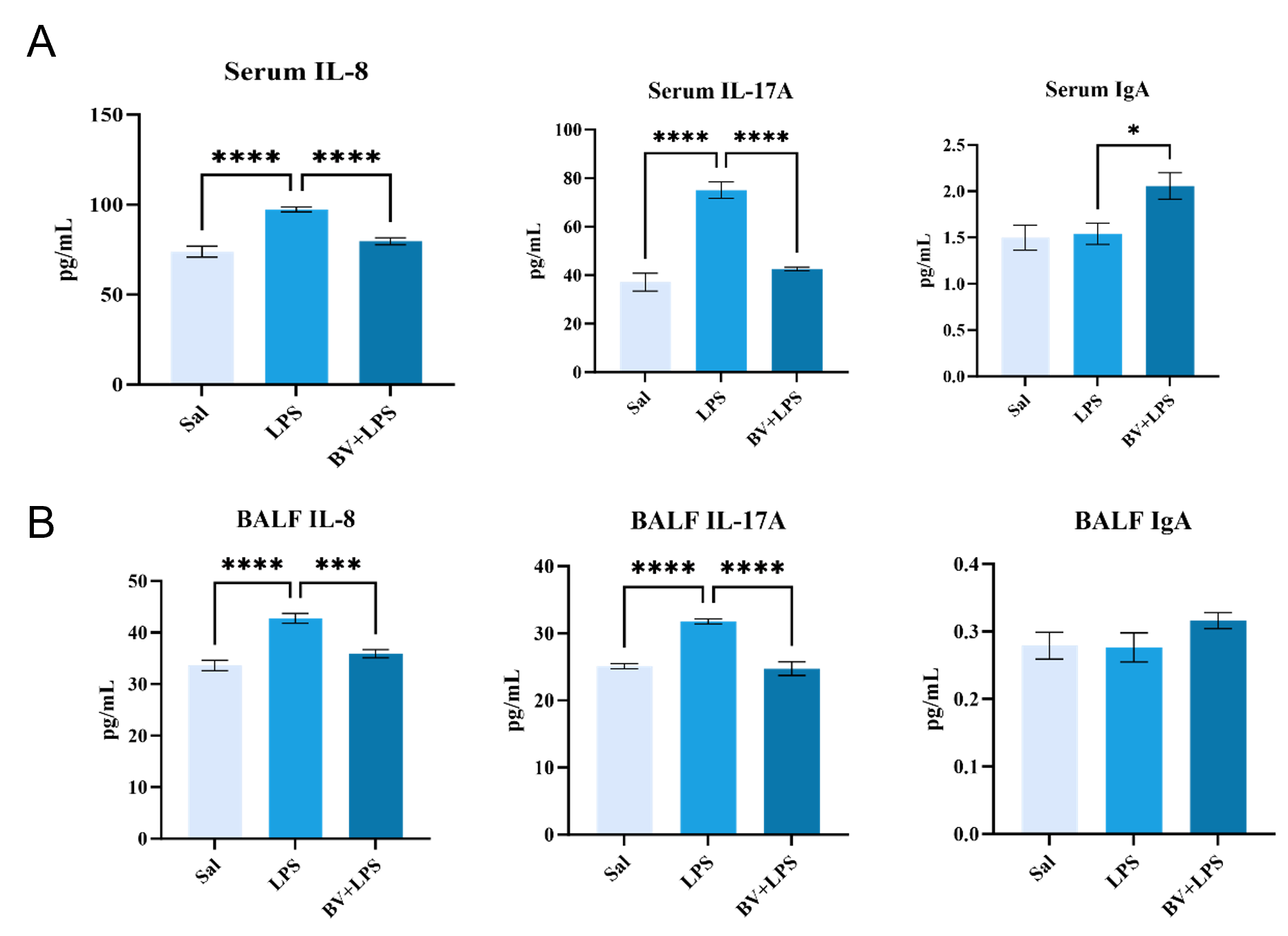


**Fig. S1** Effects of BV supplementation on inflammatory cytokine levels in broiler serum and BALF (*n*  =  6). (A) Levels of inflammatory cytokines in broiler serum. (B) Levels of inflammatory cytokines in broiler BALF.


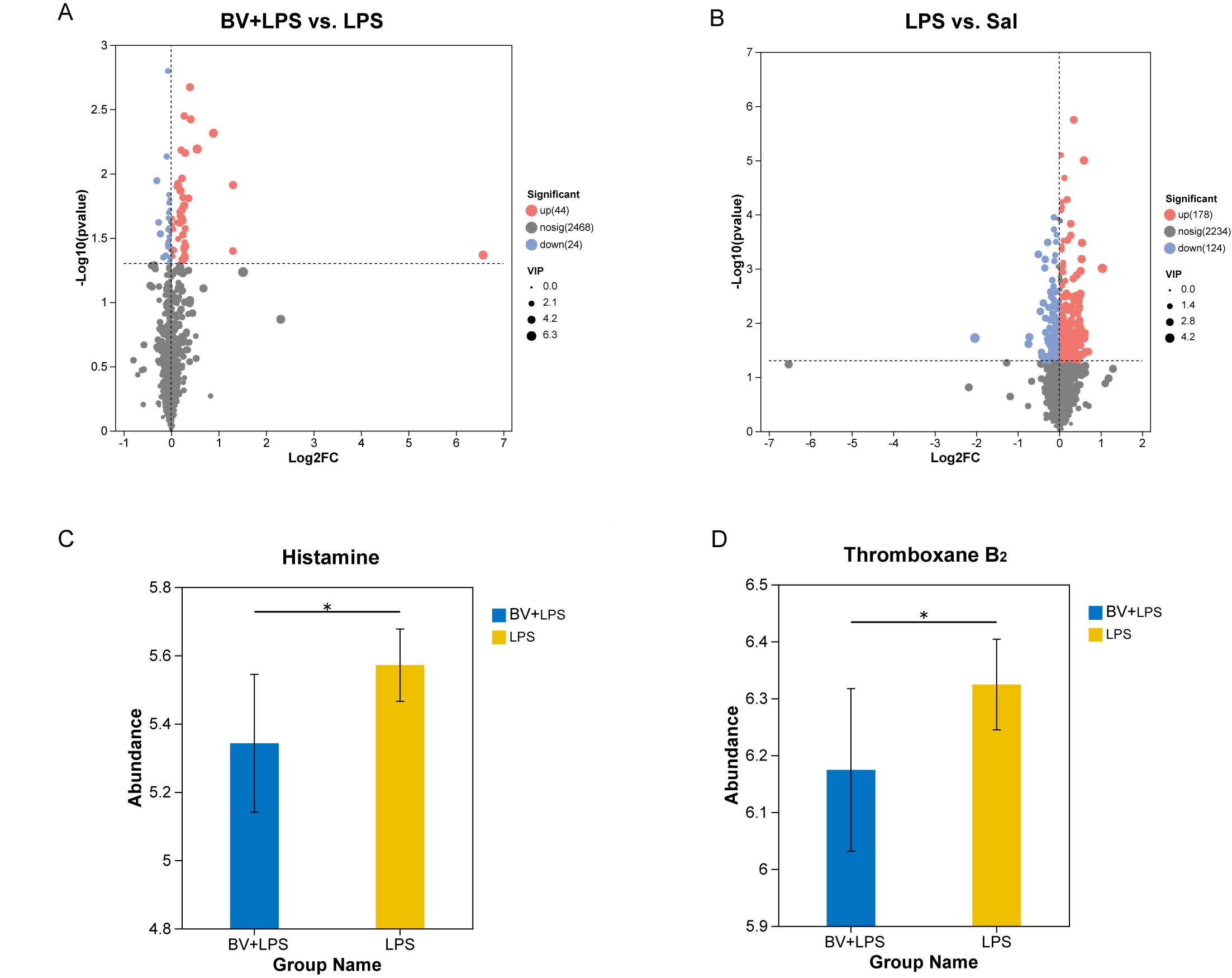


**Fig. S2** Metabolic reprogramming in broiler lungs following different treatments (*n* =  6). Volcano plots identifying differentially abundant metabolites (DAMs) between (A) BV + LPS and LPS groups; (B) LPS and Saline groups; (C) Levels of histamine; (D) thromboxane B_2_.


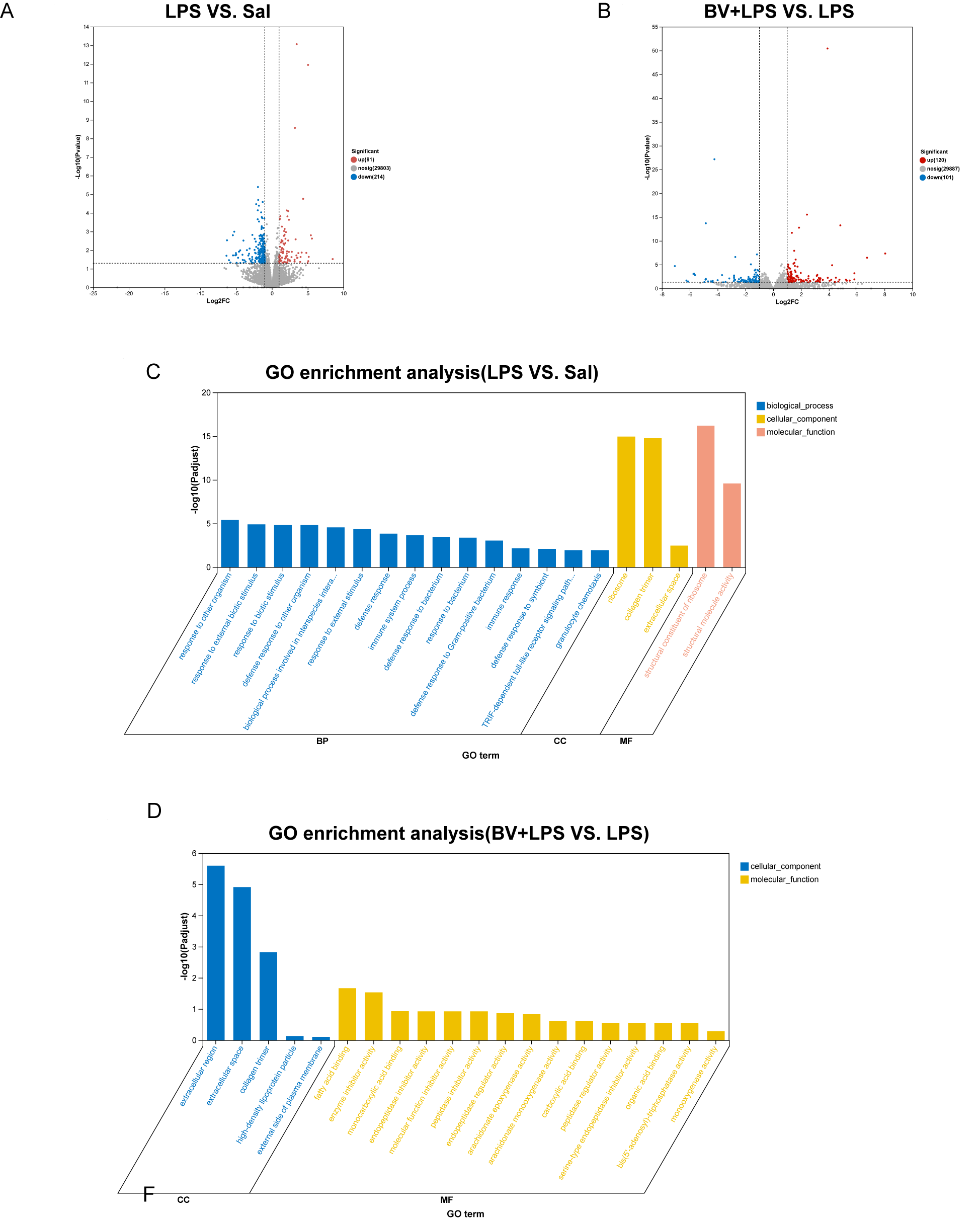


**Fig. S3** Transcriptomic analysis of broiler lungs. (A) Volcano plot of DEGs in the BV + LPS group vs. the LPS group (*n* =  3). (B) Volcano plot of DEGs in the LPS group vs. the Sal group. (C) GO enrichment analysis of DEGs from LPS vs. Sal group. (D) GO enrichment analysis of DEGs from the BV + LPS vs. LPS group.


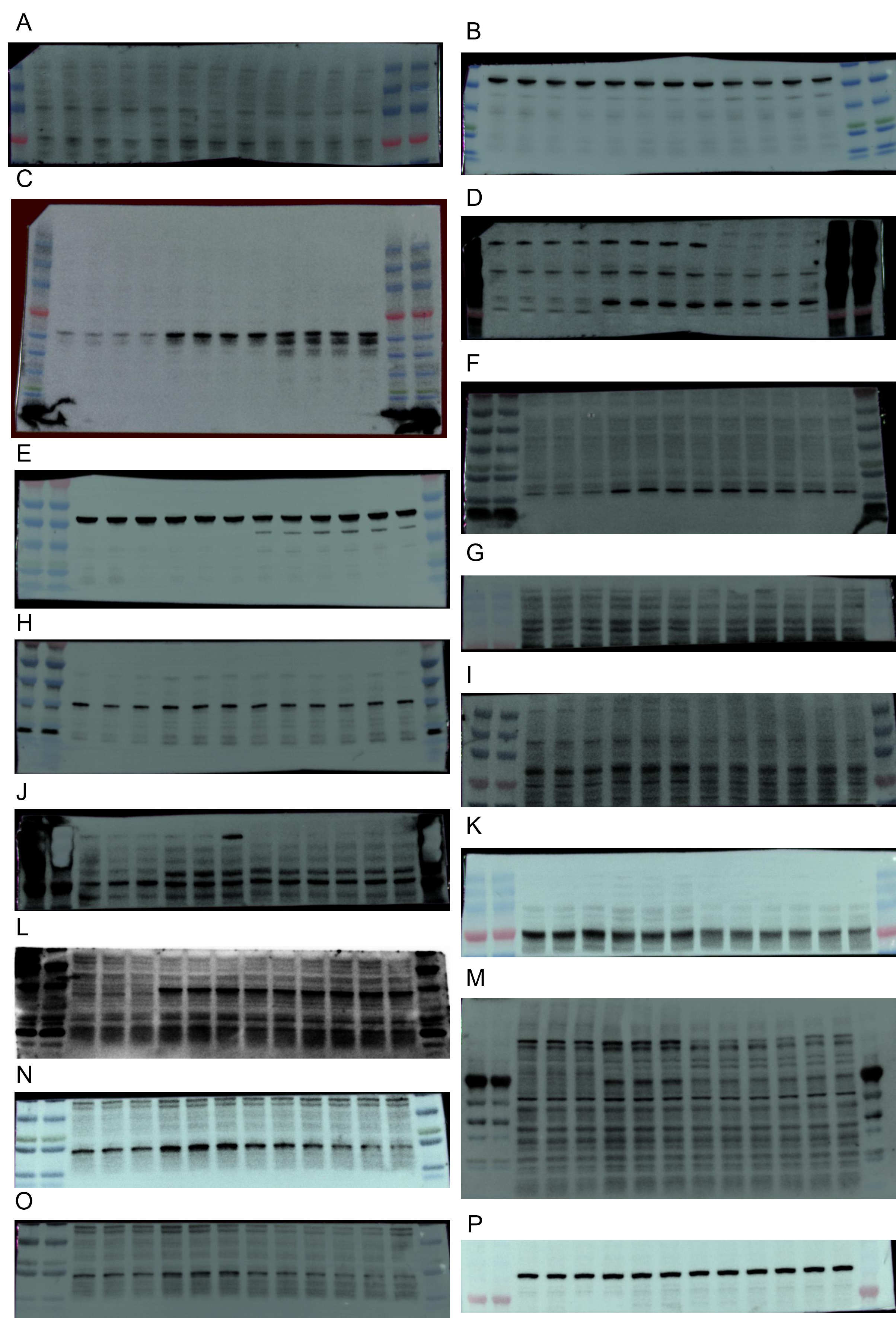


**Fig. S4** Uncropped PVDF membranes for Western blot analysis of broiler lung tissues and HD11 cells. Broiler lung: (A) p65, (B) β-actin, (C) PPAR-γ, (D) TLR4. (E) β-actin, (F) caspase-1, (G) NLRP3, (H) IL-1β, (I) CD86, (J) TLR4, (K) p65, (L) IκBα, (M) p-p65, (N) IL-10, (O) ASC, (P) CD36.


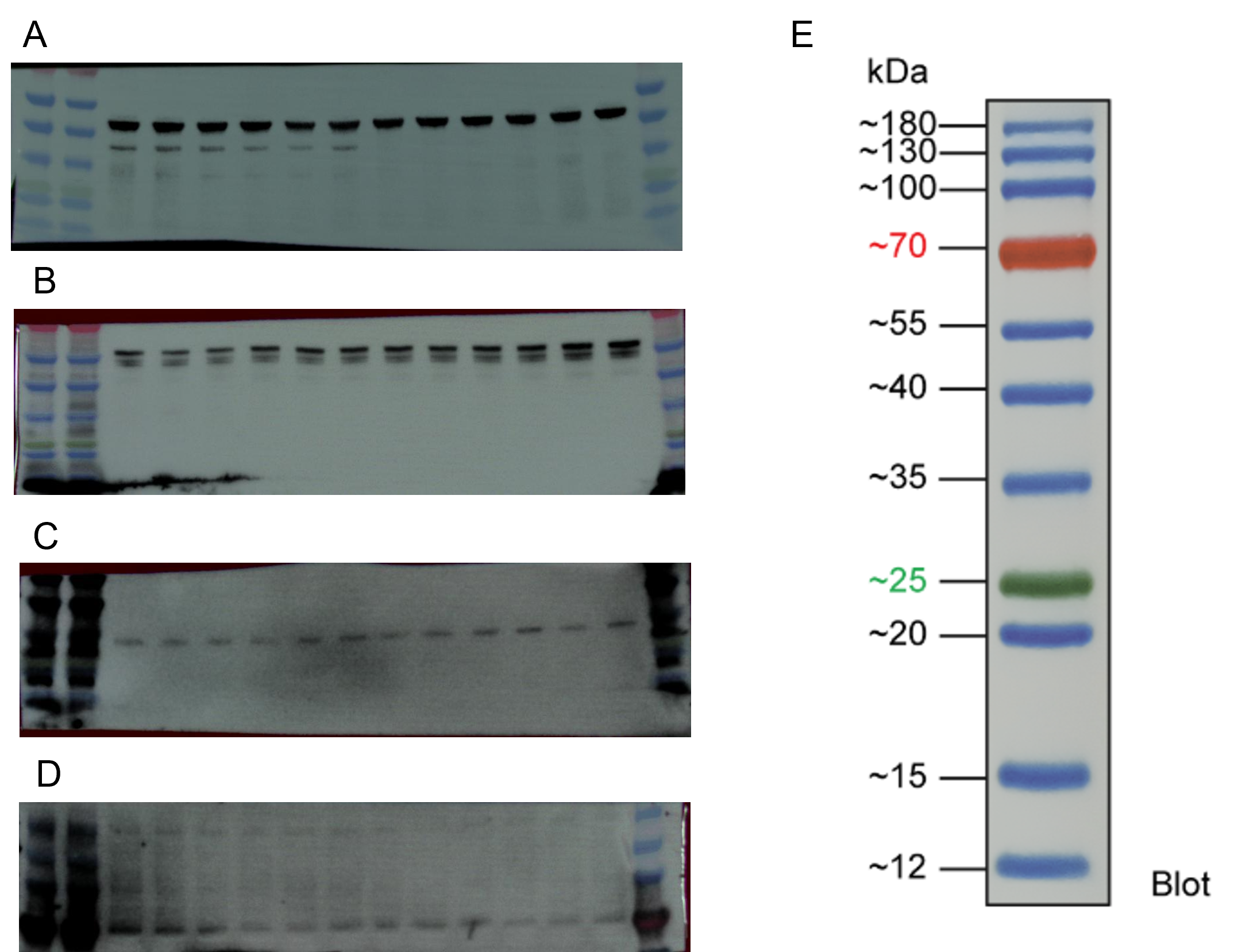


**Fig. S5** Uncropped PVDF membranes for Western blot analysis of isoflavone-treated HD11 cells. (A) β-actin, (B) PPAR-γ, (C) TLR4, (D) p65, (E) protein marker.
